# Synergistic Combination of Sb_2_Si_2_Te_6_ Additives for Enhanced Average ZT and Single‐Leg Device Efficiency of Bi_0.4_Sb_1.6_Te_3_‐based Composites

**DOI:** 10.1002/advs.202400870

**Published:** 2024-03-29

**Authors:** Xian Yi Tan, Jinfeng Dong, Jiawei Liu, Danwei Zhang, Samantha Faye Duran Solco, Kıvanç Sağlık, Ning Jia, Ivan Joel Wen Jie You, Sheau Wei Chien, Xizu Wang, Lei Hu, Yubo Luo, Yun Zheng, Debbie Xiang Yun Soo, Rong Ji, Ken Choon Hwa Goh, Yilin Jiang, Jing‐Feng Li, Ady Suwardi, Qiang Zhu, Jianwei Xu, Qingyu Yan

**Affiliations:** ^1^ Institute of Materials Research and Engineering (IMRE) Agency for Science Technology and Research (A*STAR) 2 Fusionopolis Way, Innovis #08‐03 Singapore 138634 Republic of Singapore; ^2^ School of Materials Science and Engineering Nanyang Technological University 50 Nanyang Ave, Block N4.1 #01‐30 Singapore 639798 Republic of Singapore; ^3^ Institute of Sustainability for Chemicals Energy and Environment (ISCE2) Agency for Science Technology and Research (A*STAR) 1 Pesek Road, Jurong Island Singapore 627833 Republic of Singapore; ^4^ Key Laboratory of Materials for High Power Laser Shanghai Institute of Optics and Fine Mechanics Chinese Academy of Sciences Shanghai 201800 P. R. China; ^5^ NUS High School of Mathematics and Science 20 Clementi Avenue 1 Singapore 117542 Republic of Singapore; ^6^ State Key Laboratory for Mechanical Behavior of Materials Xi'an Jiaotong University Xi'an 710049 P. R. China; ^7^ State Key Laboratory of Materials Processing and Die & Mould Technology School of Materials Science and Engineering Huazhong University of Science and Technology Wuhan 430074 P. R. China; ^8^ Key Laboratory of Optoelectronic Chemical Materials and Devices Ministry of Education Jianghan University Wuhan 430056 P. R. China; ^9^ State Key Laboratory of New Ceramics and Fine Processing School of Materials Science and Engineering Tsinghua University Beijing 100084 China; ^10^ Department of Electronic Engineering The Chinese University of Hong Kong Shatin, New Territories Hong Kong 999077 China; ^11^ School of Chemistry Chemical Engineering and Biotechnology Nanyang Technological University 21 Nanyang Link Singapore 637371 Republic of Singapore; ^12^ Department of Chemistry National University of Singapore 3 Science Drive 3 Singapore 117543 Republic of Singapore

**Keywords:** antimony silicon telluride, bismuth antimony telluride, energy harvesting, nanocomposites, Sb_2_Si_2_Te_6_, thermoelectric materials

## Abstract

Thermoelectric materials are highly promising for waste heat harvesting. Although thermoelectric materials research has expanded over the years, bismuth telluride‐based alloys are still the best for near‐room‐temperature applications. In this work, a ≈38% enhancement of the average *ZT* (300−473 K) to 1.21 is achieved by mixing Bi_0.4_Sb_1.6_Te_3_ with an emerging thermoelectric material Sb_2_Si_2_Te_6_, which is significantly higher than that of most Bi_y_Sb_2−y_Te_3_‐based composites. This enhancement is facilitated by the unique interface region between the Bi_0.4_Sb_1.6_Te_3_ matrix and Sb_2_Si_2_Te_6_‐based precipitates with an orderly atomic arrangement, which promotes the transport of charge carriers with minimal scattering, overcoming a common factor that is limiting *ZT* enhancement in such composites. At the same time, high‐density dislocations in the same region can effectively scatter the phonons, decoupling the electron‐phonon transport. This results in a ≈56% enhancement of the thermoelectric quality factor at 373 K, from 0.41 for the pristine sample to 0.64 for the composite sample. A single‐leg device is fabricated with a high efficiency of 5.4% at Δ*T* = 164 K further demonstrating the efficacy of the Sb_2_Si_2_Te_6_ compositing strategy and the importance of the precipitate‐matrix interface microstructure in improving the performance of materials for relatively low‐temperature applications.

## Graphical abstract

By mixing Bi_0.4_Sb_1.6_Te_3_ with an emerging thermoelectric material Sb_2_Si_2_Te_6_, the resulting interfaces possess an orderly arrangement with high‐density dislocations, facilitating hole transport while scattering phonons. Through the decoupling of charge‐phonon transport, the Bi_0.4_Sb_1.6_Te_3_ +1% Sb_2_Si_2_Te_6_ composite achieved a high average *ZT* (300−473 K) of 1.21 and a single‐leg device efficiency of 5.4% at Δ*T* = 164 K.

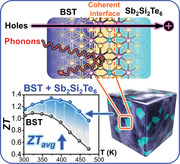

## 1 Introduction

With as much as 20 – 50% of energy constantly being lost as waste heat from automobiles, powerplants, incinerators, and other industrial processes,^[^ ^1^ ^]^ waste heat recovery through the usage of thermoelectric (TE) devices has become an attractive opportunity to conserve energy in these processes. TE devices are solid‐state devices that directly convert heat to electricity, where their conversion efficiencies are usually evaluated by the thermoelectric figure of merit, *ZT* = *S* ^2^·*σ*·*T*/*κ*. From the given equation, an efficient thermoelectric material requires a high Seebeck coefficient (S) to maintain a high potential difference across an applied thermal gradient; a high electrical conductivity (*σ*) for an efficient flow of charges; and a low total thermal conductivity (*κ*) to minimize thermal shorting. The numerator *S^2^·σ* in the ZT equation is also known as the thermoelectric power factor (PF), while the denominator can be further expressed as *κ* = *κ*_e_ + *κ*_L_, considering the electronic (*κ*_e_) and lattice (*κ*_L_) contributions. However, improving the ZT is not as straightforward as the equation depicts, because the three main performance parameters (S, σ, *κ*) are adversely correlated, thus placing constraints on the maximum achievable ZT.

Thermoelectric materials can generally be classified according to their optimum application temperature ranges.^[^ ^2^ ^]^ High‐temperature (900 – 1200 K) thermoelectric materials, such as Half‐Heusler alloys,^[^ ^3^ ^]^ silicon‐based materials,^[^ ^4^ ^]^ and oxides,^[^ ^5^ ^]^ are more tailored to be used in radioisotope thermoelectric generators to power deep‐space probes. AgSbTe_2_^[^ ^6^ ^]^ and GeTe^[^ ^7^ ^]^ are medium‐temperature (600 – 900 K) thermoelectric materials that have been gaining increasing popularity in recent years as advancements in the understanding of electrical and phonon band structure engineering have shed light on the untapped potential of these materials. Besides the studies on the materials in their traditional bulk form, there have also been interesting reports on fabricating thermoelectric materials and devices by additive manufacturing,^[^ ^8^ ^]^ thin film deposition,^[^ ^9^ ^]^ or even in their nanoscale forms.^[^ ^10^ ^]^

However, for near‐room‐temperature applications, Bi_y_Sb_2−y_Te_3_‐based materials remain the dominant choice and are the only materials used for current commercial Peltier cooler modules, with many studies on improving and evaluating their mechanical performance and thermal stability for practical device applications.^[^ ^2^, ^11^ ^]^ Various processing techniques have been used to improve the *ZT* of polycrystalline Bi_y_Sb_2−y_Te_3_‐based materials, often through the enhancement of phonon scattering to reduce the *κ*_L_. Such strategies include dislocation defect engineering by excess liquid Te‐assisted sintering,^[^ ^12^ ^]^ controlled porosity,^[^ ^13^ ^]^ swapped bilayer defects,^[^ ^14^ ^]^ twin boundary engineering,^[^ ^13^, ^15^ ^]^ and melt‐spinning.^[^ ^16^ ^]^ Another popular strategy would be the formation of nanocomposite pellet materials through the deliberate mixture of secondary phase additive materials into the Bi_y_Sb_2−y_Te_3_ matrix material. Notable secondary phase additive materials used for such purposes include graphene,^[^ ^17^ ^]^ diamond,^[^ ^18^ ^]^ ZnO,^[^ ^19^ ^]^ SiC,^[^ ^20^ ^]^ and even organometallic molecules like copper(II) phthalocyanine.^[^ ^21^ ^]^ These additives typically enhance the *ZT* of Bi_y_Sb_2−y_Te_3_‐based materials by reducing the lattice thermal conductivity and tuning the charge carrier concentration, even serving to improve the mechanical properties at the same time. The plethora of possible secondary phase additives for mixing with Bi_y_Sb_2−y_Te_3_‐based materials is virtually limitless, demonstrating the versatility of this strategy and a great space for further exploration.

However, for most of these composites, the lattice thermal conductivity reduction is also accompanied by a significant deterioration of their carrier mobilities,^[^ ^17^, ^18^, ^19^, ^21^, ^22^ ^]^ which is usually due to the formation of abrupt interfaces that strongly scatter charge carriers. Given the relatively high mobility of pristine Bi_y_Sb_2−y_Te_3_ alloys (192 – 340 cm^2^ V^−1^ s^−1^),^[^ ^23^ ^]^ the detrimental effect of charge carrier scattering is especially pronounced in Bi_y_Sb_2−y_Te_3_‐based composites. Since the degree of *ZT* enhancement of the Bi_y_Sb_2−y_Te_3_‐based composites is largely limited by the charge carrier scattering effects, there is a clear demand for new types of additives or compositing strategies that can overcome this bottleneck in order for the advancement of Bi_y_Sb_2−y_Te_3_‐based composites to ascend toward its full potential.

One such promising additive in consideration is Sb_2_Si_2_Te_6_, with a low room‐temperature thermal conductivity of 1.2 – 1.4 W m^−1^K^−1^, which can continue to decrease monotonically when temperatures are increased until 823 K.^[^ ^24^ ^]^ Besides its low thermal conductivity, it also has a valence band energy level of −4.86 eV (w.r.t. vacuum level),^[^ ^24^ ^]^ which is very close to the valence band energy level of Bi_0.4_Sb_1.6_Te_3_ (−4.926 eV w.r.t. vacuum level).^[^ ^25^ ^]^ In addition, both Bi_0.4_Sb_1.6_Te_3_ and Sb_2_Si_2_Te_6_ can be seen as derivations of Sb_2_Te_3_‐based materials with layered structures and rhombohedral symmetries, which increases the chances of forming a coherent interface between them. Such a close valence band alignment between the two dissimilar material phases and the possibility of forming coherent interfaces may imply a minimal disturbance to the hole transport properties, potentially overcoming the *ZT* enhancement limits caused by detrimental charge carrier scattering. Coupled with phonon scattering at the interfaces, this is a promising strategy that may lead to an enhanced *ZT*.

Sb_2_Si_2_Te_6_ is itself a promising medium‐temperature p‐type TE material and also an emerging thermoelectric material phase that was first reported in 2020,^[^ ^24^ ^]^ and shortlisted as one of the promising new materials that fulfill the general selection rules for high *ZT*s over a wide temperature range.^[^ ^26^ ^]^ Some of its key promising features include its layered 2D crystal structure that favors a low intrinsic lattice thermal conductivity (*κ*_L_) due to its high Grüneisen parameter, low‐lying optical phonon mode, and low maximum acoustic phonon mode frequency. This low intrinsic *κ*_L_ can be reduced even further in the final *κ*_L_ by introducing microstructural defects. Combined with a decent PF, pristine Sb_2_Si_2_Te_6_ can achieve a high *ZT* of 1.08 at 550 °C.^[^ ^24^ ^]^ While there have been some other recent works on exploring the thermoelectric properties of (Sb,Bi)_2_Si_2_Te_6_‐based materials mostly through substitutional doping using Bi,^[^ ^27^ ^]^ Ca,^[^ ^28^ ^]^ or Ge,^[^ ^29^ ^]^ to the best of our knowledge, there is a clear lack of research works involving the deliberate mixing of additive materials into (Sb,Bi)_2_Si_2_Te_6_‐based materials, and much less on using (Sb,Bi)_2_Si_2_Te_6_‐based materials themselves as additive materials to be mixed into other materials for thermoelectric applications.

In this work, we used Sb_2_Si_2_Te_6_ as an additive in the Bi_0.4_Sb_1.6_Te_3_ matrix phase to produce Bi_0.4_Sb_1.6_Te_3_ + *x* mol% Sb_2_Si_2_Te_6_ composite materials, for *x* = 0.5, 1, 2, 3, and 4%, in order to explore its effects on the thermoelectric properties. Pristine Bi_0.4_Sb_1.6_Te_3_ and Sb_2_Si_2_Te_6_ ingots were first synthesized and pulverized into fine powders separately before homogeneously mixing the two powders in various proportions by ball milling, before sintering the powder mixture into dense bulk pellets. The resulting *x* = 1% composite pellet was found to have semi‐coherent interfaces with a high density of dislocations, between the Sb_2_Si_2_Te_6_‐based precipitate and the Bi_0.4_Sb_1.6_Te_3_ matrix, which helps scatter phonons with the negligible compromise of carrier transport. Such a decoupled modulation of carrier and thermal transport led to an enhanced power factor, reduced lattice thermal conductivity, and an improved average *ZT* of 1.21 over a temperature range of 300 to 473 K. The effectiveness of the Sb_2_Si_2_Te_6_ compositing strategy was proven again with a high single‐leg device efficiency of 5.4% achieved by the *x* = 1% sample at Δ*T* = 164 K.

## 2 Results and Discussion

### 2.1 Structural Characterization

**Figure 1:**
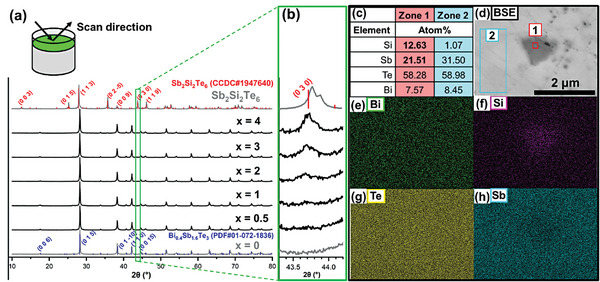
Powder X‐ray diffractograms of Bi_0.4_Sb_1.6_Te_3_ + *x* mol% Sb_2_Si_2_Te_6_ pellet samples for varying amounts of *x*, scanned at a) 2*θ* = 10 – 80° and b) 2*θ* = 43.3 – 44.2°. c) Elemental composition and d) Backscattered Electron (BSE) image of a secondary phase precipitate in the *x* = 1% pellet sample by Scanning Electron Microscopy (SEM). Elemental maps showing the distribution of e) Bi, f) Si, g) Te, and h) Sb.

The overall phase purities of the Bi_0.4_Sb_1.6_Te_3_ + *x* mol% Sb_2_Si_2_Te_6_ samples were measured by Powder X‐ray Diffraction (XRD) analysis, with their diffractograms plotted as shown in **Figure** **1**a. While all samples can be well‐indexed to the Bi_0.4_Sb_1.6_Te_3_ phase (PDF# 01‐072‐1836), the emergence of secondary phase peaks at 2*θ* = 35.6° and 43.7° were observed to be increasing in intensity from *x* = 2% to *x* = 4%, where the increasing intensity of the later peak can be clearly seen in the high‐resolution scan range of 2*θ* = 43.3 – 44.2° in Figure 1b. These secondary phase peaks were found to match the (02$\bar{5}$) and (030) peaks of Sb_2_Si_2_Te_6_ (CCDC# 1947640) respectively. Further investigation of the *x* = 1% pellet sample by Scanning Electron Microscopy (SEM) revealed the presence of secondary phase particles, where the darker particle in the Backscattered Electron (BSE) image of Figure 1d was found to have a size of ≈1 µm. Upon analysis by Energy Dispersive Spectroscopy (EDS) and elemental mapping as shown in Figure 1e–h, the particle (Zone 1) was found to be much richer in Si and slightly more deficient in Sb as compared to the surrounding matrix (Zone 2), which supports the conclusion of the presence of Sb_2_Si_2_Te_6_ precipitates, since Bi_0.4_Sb_1.6_Te_3_ has a Sb content of 32% while Sb_2_Si_2_Te_6_ has a lower Sb content of 20%. From Figure S1 (Supporting Information), the secondary phase micro‐precipitates appeared to be rather homogeneously distributed throughout the *x* = 1% sample, as depicted in the SEM elemental maps at lower magnifications. This may be due to the ball milling process used to pulverize the ingots and homogenize the powder mixtures, which also contributed to a rather small average grain size of ≈2 µm, based on the SEM image of the pellet's fractured morphology in Figure S2b (Supporting Information).

**Figure 2:**
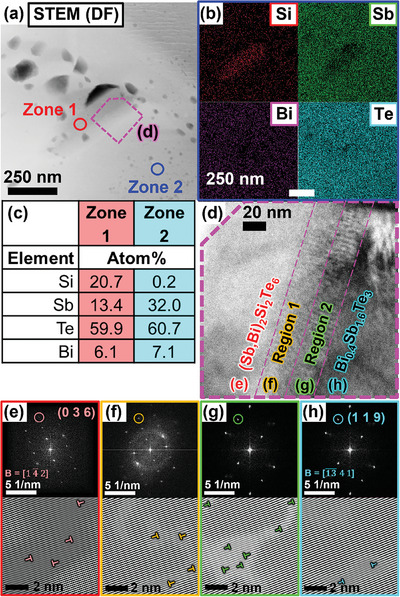
a) Low magnification dark‐field STEM image of the *x* = 1% pellet sample. b) Elemental maps showing the distribution of Si, Sb, Bi, and Te. c) EDS elemental composition of the red and blue circled zones indicated in a). d) HRTEM image of the magenta‐bordered area marked in a), where the three distinct regions of (Sb,Bi)_2_Si_2_Te_6_, the interface regions, and Bi_0.4_Sb_1.6_Te_3_ can be seen. Fast Fourier Transformation (FFT) patterns with Inverse FFT images of the circled points, for e) (Sb,Bi)_2_Si_2_Te_6_, f) Interface Region 1, g) Interface Region 2, and h) Bi_0.4_Sb_1.6_Te_3_. Dislocations are marked with “┴” symbols in the Inverse FFT images.

Besides the micro‐precipitates, a nanoscale secondary phase precipitate (≈300 nm long) was also found in the *x* = 1% pellet sample, as shown in the Scanning Transmission Electron Microscopy (STEM) image (**Figure** **2**a). Based on the EDS elemental composition from the inset table in Figure 2c, together with the elemental maps shown in Figure 2b, the nanoprecipitate is most likely Sb_2_Si_2_Te_6_ with Bi_Sb_^×^ substitutional defects, which will be referred to as “(Sb,Bi)_2_Si_2_Te_6_”. The (Sb,Bi)_2_Si_2_Te_6_ nanoprecipitate appears as a darker region in the dark field STEM image due to their lower average molecular weights, as compared to the surrounding Bi_0.4_Sb_1.6_Te_3_ matrix. Comparing the elemental compositions of the nanoprecipitate and matrix in Figures 1c and 2c, as well as the EDS line scan profile in Figure S3 (Supporting Information), it was evident that the Bi composition in the nanoprecipitate and the matrix are relatively similar, indicating a rather homogeneous diffusion of Bi throughout the composite. In addition, the lattice constants of all samples obtained by Rietveld refinement of their XRD patterns showed a slight decrease with increasing Sb_2_Si_2_Te_6_ content, as demonstrated in Figure S4a (Supporting Information). While this decrease in lattice constants was not very significant (≈0.01 Å decrease from *x* = 0% to *x* = 4%) due to the small amounts of Sb_2_Si_2_Te_6_ precipitates added, this still implies that there is a slightly higher proportion of the smaller Sb^3+^ ions remaining in the Bi_0.4_Sb_1.6_Te_3_ matrix, due to a depletion of the bigger Bi^3+^ ions as they get diffused into the Sb_2_Si_2_Te_6_ precipitates. The gradual increase of Si and reduction of Sb content from the surrounding matrix to the nanoprecipitate from the line scan profile in Figure S3 (Supporting Information) also implies the presence of a ≈50 nm thick diffusion layer at the interface between the two phases.

In order to investigate the interface region between the (Sb,Bi)_2_Si_2_Te_6_ nanoprecipitate and the surrounding Bi_0.4_Sb_1.6_Te_3_ matrix, a bright field High‐Resolution Transmission Electron Microscopy (HRTEM) image was captured (Figure 2d). As labeled in Figure 2d, HRTEM images with high magnifications were taken for each of the four regions, as shown in Figure S5d – g (Supporting Information). From each of their magnified HRTEM images, Fast Fourier Transformation (FFT) was performed, with the electron diffraction patterns of the (Sb,Bi)_2_Si_2_Te_6_, Interface Region 1, Interface Region 2, and Bi_0.4_Sb_1.6_Te_3_ regions displayed in Figure 2e – h, respectively. The diffraction pattern of the nanoprecipitate (Figure 2e) can be indexed to the rhombohedral structure of Sb_2_Si_2_Te_6_ (CCDC# 1947640) with a zone axis B = [1 $\bar{4}$ 2], demonstrating the preservation of the Sb_2_Si_2_Te_6_ crystal structure without undergoing decomposition. The diffraction pattern of the matrix (Figure 2h) can be indexed to the rhombohedral structure of Bi_0.4_Sb_1.6_Te_3_ (PDF# 01‐072‐1836) with a zone axis B = [$\bar{13}$ 4 1], where all its 9 points can also be found in very similar positions on the diffraction pattern of the nanoprecipitate (Figure 2e). Such similar crystal structures of the (Sb,Bi)_2_Si_2_Te_6_ nanoprecipitate and the Bi_0.4_Sb_1.6_Te_3_ matrix can lead to a high site coincidence and a semi‐coherent interface. This can also facilitate the diffusion of atoms and the formation of the thick diffusion layer observed between the two phases, which were divided into “Interface Regions 1 and 2”, where each of their electron diffraction patterns were analyzed separately as Figure 2g,h, respectively. Interface Regions 1 and 2 were observed to have single crystal‐like diffraction patterns which are nearly identical to those of the (Sb,Bi)_2_Si_2_Te_6_ nanoprecipitate and the Bi_0.4_Sb_1.6_Te_3_ matrix respectively, indicating that the atoms in the interface region occupy an orderly arrangement. Such an orderly interface region can minimize its influence on electron transport, thus securing a high charge carrier mobility. For a fair comparison, a circled point on a position common to all four electron diffraction patterns from Figure 2e – h was then used to generate the Inverse FFT images. While the Bi_0.4_Sb_1.6_Te_3_ matrix showed a rather perfect atomic arrangement with little dislocations (Figure 2h), the inverse FFT images of the (Sb,Bi)_2_Si_2_Te_6_ nanoprecipitate and the interface regions revealed a much higher density of dislocations, which can be beneficial for phonon scattering in the mid‐frequency range.

Based on the above SEM and TEM observations in Figures 1d and 2a, Figures S2a,S1, and S5a, (Supporting Information) we could conclude that the average sizes of Sb_2_Si_2_Te_6_ precipitates and Bi_0.4_Sb_1.6_Te_3_ matrix were a few hundred nm and a few microns, respectively. The relatively small size of the secondary phase is expected for a composite, which would lead to a higher total interface area with the matrix phase. According to the similar electron diffraction patterns of Sb_2_Si_2_Te_6_ precipitates, the matrix, and the interface, a coherent/semi‐coherent interface was formed between them, which can be expected due to their similar crystal structures. Such coherent/semi‐coherent interfaces can facilitate the transport of electrons, maintaining high carrier mobility as discussed later. At the same time, the mass mismatching between the Sb_2_Si_2_Te_6_ precipitates and Bi_0.4_Sb_1.6_Te_3_ matrix, as well as the dislocations at the interface region, would scatter phonons to decrease the lattice thermal conductivities.

### 2.2 Charge Transport Properties

**Figure 3:**
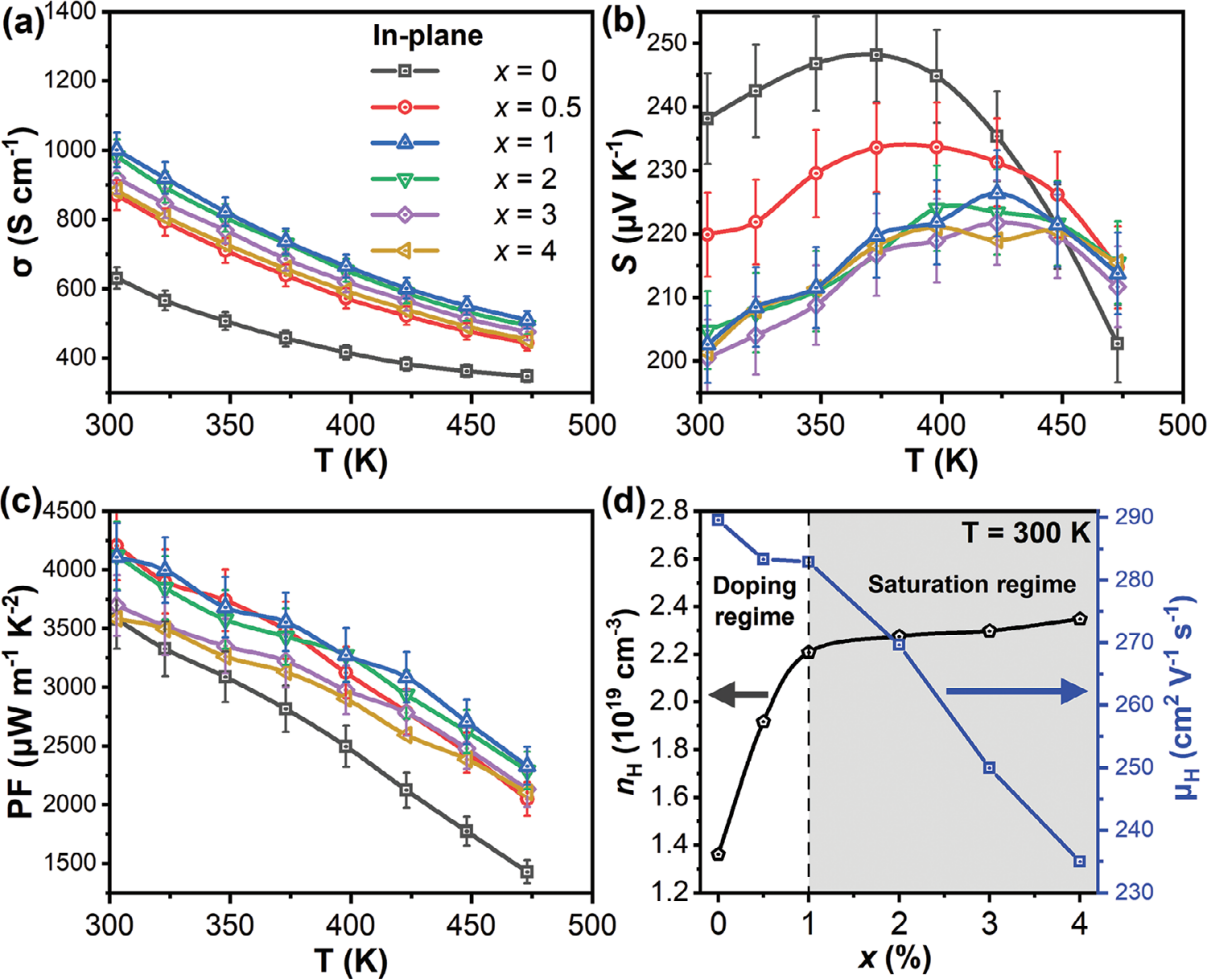
Temperature‐dependent a) electrical conductivities (σ), b) Seebeck coefficients (*S*), and c) thermoelectric Power Factors (PF) of the composite samples, for various Sb_2_Si_2_Te_6_ contents. d) Composition (*x*)‐dependent hole concentrations (*n*_H_) and hole mobilities (μ_H_) obtained by Hall effect measurements at room temperature.

From **Figure** **3**a,b, increasing the Sb_2_Si_2_Te_6_ ratio “*x*” from 0 to 1% resulted in an increasing trend in electrical conductivity and a decreasing trend in the Seebeck coefficient. The trends can be explained by the increase in hole concentration observed in Figure 3d with increasing Sb_2_Si_2_Te_6_ content. This doping effect is expected considering the higher hole concentration of pristine Sb_2_Si_2_Te_6_ (5.6 × 10^19^ – 7.1 × 10^19^ cm^−3^),^[^ ^24^, ^27^ ^]^ as compared to that of pristine Bi_0.4_Sb_1.6_Te_3_ (1.26 × 10^19^ − 2.7 × 10^19^ cm^−3^).^[^ ^23^ ^]^ Besides direct carrier injection from the nanoprecipitates, the increase in hole concentration could also be due to the depletion of Bi atoms from the Bi_0.4_Sb_1.6_Te_3_ matrix when the Bi diffused evenly into all Sb_2_Si_2_Te_6_ nanoprecipitates to form (Sb,Bi)_2_Si_2_Te_6_ (Figure 2c; Figure S3, Supporting Information), as reducing *y* in Bi_y_Sb_2−y_Te_3_‐based materials has been known to increase their hole concentrations.^[^ ^30^ ^]^ Due to the much lower reported bulk hole mobility of Sb_2_Si_2_Te_6_ (30 – 58 cm^2^ V^−1^s^−1^),^[^ ^24^, ^27^ ^]^ as compared to Bi_0.4_Sb_1.6_Te_3_ (192 – 340 cm^2^ V^−1^s^−1^),^[^ ^23^ ^]^ a reduction in hole mobility was expected. However, the reduction in hole mobility from *x* = 0 to 1% is relatively small, maintaining a high value of >280 cm^2^ V^−1^s^−1^. This can be attributed to the ordered atomic arrangement of the interface region observed in Figure 2e – h and the valence band alignment between Bi_0.4_Sb_1.6_Te_3_ and Sb_2_Si_2_Te_6_. A coherent/semi‐coherent interface prevents the breakdown of the bonds and maintains the transport path for carriers. Furthermore, the energy difference of the valence band maxima (VBM) between Bi_0.4_Sb_1.6_Te_3_ and Sb_2_Si_2_Te_6_ is only 0.06 eV (Figure S6, Supporting Information).^[^ ^24^, ^25^ ^]^ Such a small energy difference indicates a valence band alignment for Bi_0.4_Sb_1.6_Te_3_ and Sb_2_Si_2_Te_6_, facilitating the transport of holes between these two phases. Overall, this increased the room‐temperature power factors of the composites from 3580 µW m^−1^ K^−2^ for *x* = 0%, to 4207 and 4109 µW m^−1^ K^−2^ for *x* = 0.5 and 1% respectively, as shown in Figure 3c.

However, from the decreasing trend in electrical conductivities from *x* = 1% to *x* = 4%, as well as the relatively similar Seebeck coefficients of the *x* = 1 − 4% samples, there seems to be a saturation point in hole donation from *x* = 1% and higher, such that the Seebeck coefficients and hole concentrations did not change significantly, as shown in Figure 3b,d. On the other hand, there was a significant reduction in hole mobilities from *x* = 1% to 4%, due to the presence of additional Sb_2_Si_2_Te_6_ beyond the saturation limit of Sb_2_Si_2_Te_6_ content for efficient hole donation, which began to scatter holes to a greater degree. As the hole scattering effects dominated the small increases in hole concentration, this caused a gradual reduction in electrical conductivities with increasing *x*. Overall, this decreased the room‐temperature power factors of the composites from 4109 µW m^−1^ K^−2^ for *x* = 1% to 3584 µW m^−1^ K^−2^ for *x* = 4%, as shown in Figure 3c.

### 2.3 Thermal Transport Properties

**Figure 4:**
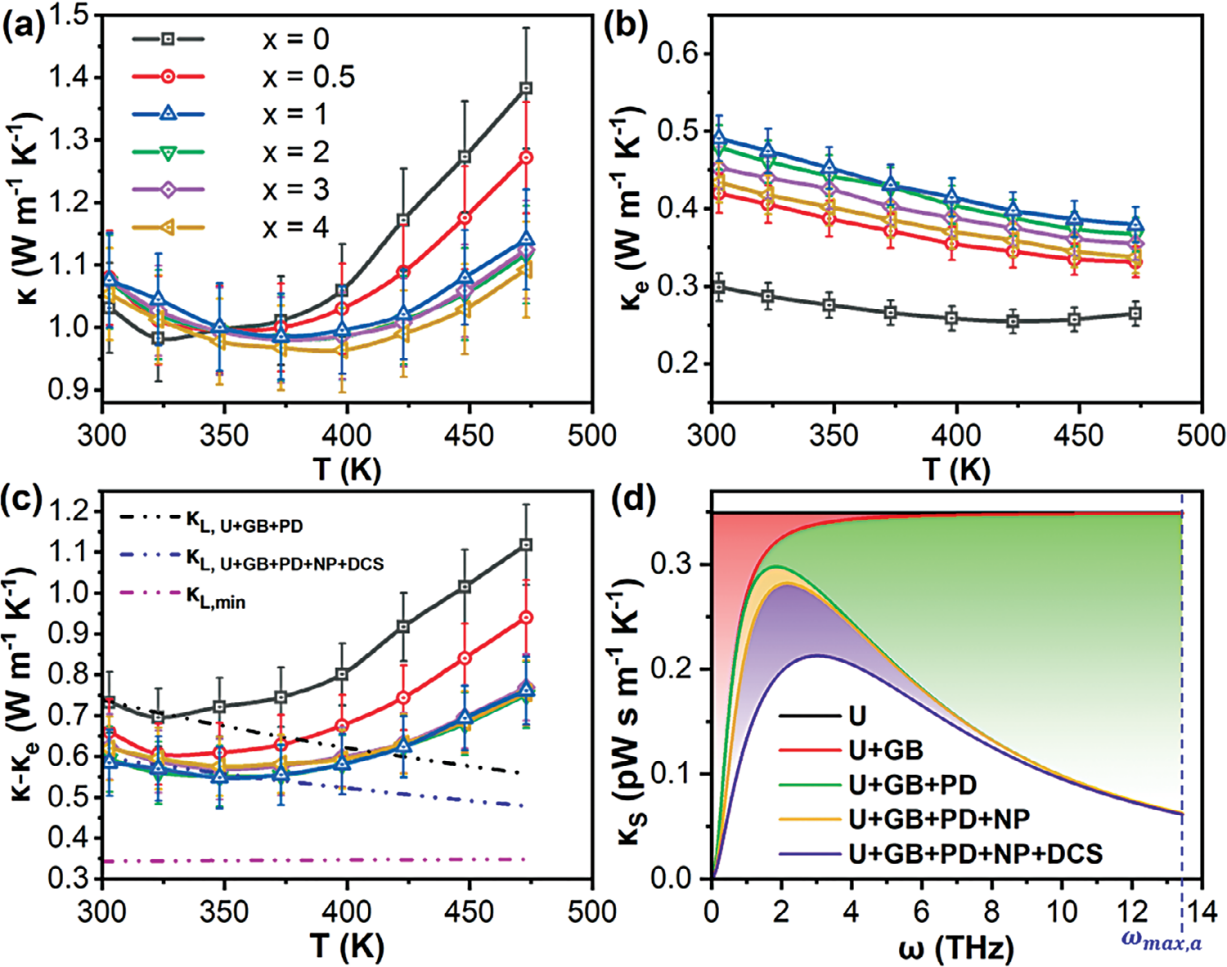
Temperature‐dependent a) total thermal conductivities (*κ*), b) electronic contributions of thermal conductivities (*κ*_e_), c) lattice and bipolar thermal conductivities (*κ* − *κ*_e_). From c), the calculated minimum lattice thermal conductivity (*κ*_L,min_), as well as the calculated lattice thermal conductivities (*κ*_L, U+GB+PD_ and *κ*_L, U+GB+PD+NP+DCS_) based on the Umklapp (U), grain boundary (GB), point defect (PD), nanoprecipitate (NP), and dislocation core and strain (DCS) phonon scattering processes are plotted as dashed lines. d) Phonon frequency (ω)‐dependent spectral thermal conductivities (*κ*_S_) showing the contributions of the various phonon scattering processes to *κ*_L_ reduction.

A slight increase in room temperature total thermal conductivities (*κ*) was observed from 1.03 W m^−1^K^−1^ in the pristine *x* = 0% sample, to 1.05 – 1.08 W m^−1^ K^−1^ for the *x* = 0.5 − 4% samples, as shown in **Figure** **4**a. This can be attributed to the significantly increased electronic contributions to the thermal conductivities (*κ*_e_) for the *x* = 0.5 − 4% samples, as shown in Figure 4b. By subtracting the electronic contribution, the resulting thermal conductivity values (*κ*‐ *κ*_e_), which chiefly consist of lattice and bipolar thermal conductivities, were plotted as shown in Figure 4c. The *κ*‐ *κ*_e_ values decreased from 0.73 W m^−1^K^−1^ in the pristine *x* = 0% sample, to 0.66 W m^−1^K^−1^ for the *x* = 0.5% sample and 0.58 – 0.62 W m^−1^ K^−1^ for the *x* = 1 − 4% samples at room temperature, demonstrating the effectiveness of mixing Sb_2_Si_2_Te_6_ in Bi_0.4_Sb_1.6_Te_3_.

Using the Debye‐Callaway model, the lattice thermal conductivity calculated based on the Umklapp (U), grain boundary (GB), and point defect (PD) phonon scattering processes (*κ*_L, U+GB+PD_) was plotted as a black dashed line in comparison with the experimental *κ*‐ *κ*_e_ values of the pristine *x* = 0% sample in Figure 4c, since the pristine Bi_0.4_Sb_1.6_Te_3_ contains grain boundaries and Bi_Sb_^×^ substitutional point defects. Upon the addition of Sb_2_Si_2_Te_6_, the presence of (Sb,Bi)_2_Si_2_Te_6_ nanoprecipitates and additional dislocations at the precipitate‐matrix interface region in the *x* = 1% sample were revealed by the STEM and the Inverse FFT images from Figure 2. Therefore, a blue dashed line calculated based on the U, GB, PD, nanoprecipitate (NP), dislocation core, and strain (DCS) phonon scattering processes (*κ*_L, U+GB+PD+NP+DCS_) was also plotted in comparison with the *x* = 1% sample's results. For the measured *κ*‐ *κ*_e_ values of the *x* = 0% and 1% samples, there is a close fit with the calculated *κ*_L, U+GB+PD_ and *κ*_L, U+GB+PD+NP+DCS_ respectively, from room temperature until the onset of bipolar conduction, which demonstrated the phonon‐scattering efficacy of the additional Sb_2_Si_2_Te_6_ nanoprecipitates. The details of the Debye‐Callaway model are explained in the Supporting Information and the input parameters used for the model are summarized in Table S1 (Supporting Information).

In order to further investigate the phonon scattering mechanisms present in the composite samples and the main reason for their reduced lattice thermal conductivities, the spectral thermal conductivities calculated using the Debye‐Callaway model were plotted against phonon frequency in Figure 4d. While zero‐dimensional PD and two‐dimensional GB scattering processes can suppress phonon transport in the high and low phonon frequency ranges respectively, the additional one‐dimensional DCS and three‐dimensional NP scattering processes that are only present upon the addition of Sb_2_Si_2_Te_6_ can scatter more phonons in the mid‐frequency ranges. Such multi‐dimensional defects present in the composite samples at all length scales are indeed beneficial for scattering phonons over the full phonon frequency range, which led to an effective reduction in the lattice thermal conductivities upon the addition of Sb_2_Si_2_Te_6_. In addition, the theoretical minimum lattice thermal conductivity (*κ*_L,min_) of Bi_0.4_Sb_1.6_Te_3_ was calculated using the Debye‐Cahill model (see Supporting Information for details) to be ≈0.34 – 0.35 W m^−1^ K^−1^ across the entire temperature range and plotted as a pink dashed line in Figure 4c. Since the room‐temperature *κ*‐ *κ*_e_ value of the best‐performing *x* = 1% sample (≈0.59 W m^−1^ K^−1^) is almost double of *κ*_L,min_, there is a lot more room for further *κ*‐ *κ*_e_ reduction.

### 2.4 Thermoelectric Performance

**Figure 5:**
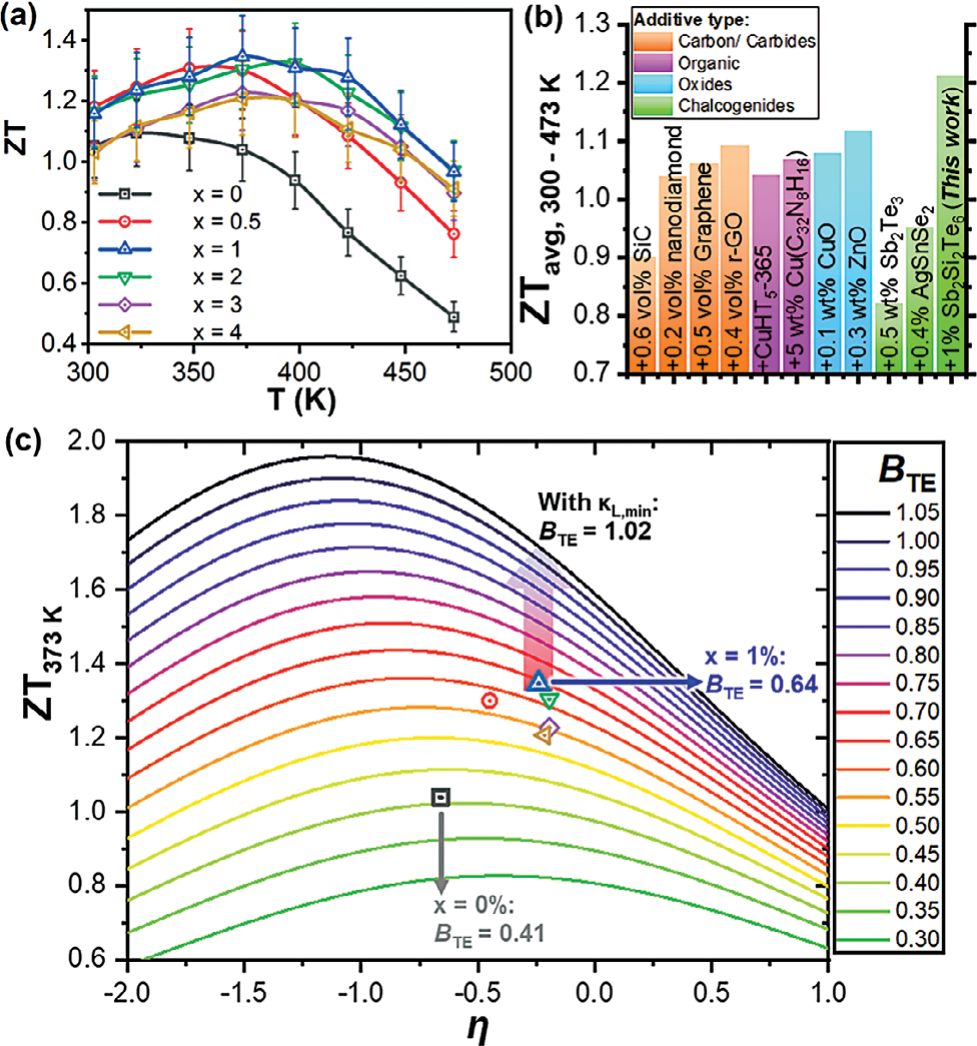
a) Temperature‐dependent thermoelectric Figures of Merit (*ZT*) of the composite samples, for various Sb_2_Si_2_Te_6_ contents. b) Average *ZT*s between T = 300 – 473 K (*ZT*_avg, 300 – 473 K_) of the other reported p‐type Bi_y_Sb_2−y_Te_3_ (BST)‐based composites with various classes of additive materials, in comparison with the *x* = 1% (BST +1% Sb_2_Si_2_Te_6_) composite sample. c) Quality factor (*B*_TE_) analysis of the composite samples at 373 K.

In general, an overall increase in the *ZT* values of Bi_0.4_Sb_1.6_Te_3_ with the addition of Sb_2_Si_2_Te_6_ can be observed across the entire temperature range, as shown in **Figure** **5**a. At a temperature of 373 K, the *ZT* increases from 1.04 to 1.35 from the pristine *x* = 0% to *x* = 1% samples. With the very similar total thermal conductivities of the *x* = 1 − 4% samples, but a much more significant decreasing trend of power factors from *x* = 1% to *x* = 4%, the peak *ZT* at 373 K decreases from 1.35 for *x* = 1%, to 1.21 for *x* = 4%. Therefore, the optimum composition with the highest peak ZT was determined to be the *x* = 1% sample, where the *ZT* of the *x* = 0.5 and 2% samples have very close values of ≈1.30 at 373 K. A progressive increase in peak ZT temperatures (*T*_peak_) was also noticeable as *x* increased from 0% (*T*_peak_ = 323 K) to 2% (*T*_peak_ = 398 K), which can be expected from the increasing hole concentration and suppression of bipolar conduction at higher temperatures. All these *ZT*s that are >1.3 from *x* = 0.5 – 2% demonstrate good reproducibility of the Sb_2_Si_2_Te_6_ additive strategy for Bi_0.4_Sb_1.6_Te_3_‐based materials, as well as a relatively forgiving composition range for optimizing the peak *ZT*. In addition, these samples are stable up to 483 K, as no evaporation or phase transition was detected (Figure S7, Supporting Information). Overall, the peak *ZT*s obtained for the *x* = 0.5 − 4% samples are all consistently significantly higher than that of the pristine *x* = 0%, which has a *ZT* of 1.04 at 373 K.

Moreover, the average *ZT* over a temperature range of 300 to 473 K (*ZT*_avg, 300 – 473 K_) was also enhanced from 0.88 for the pristine sample to a rather high value of 1.21 for the *x* = 1% sample. Besides having high peak *ZT*s, the high *ZT*s at lower temperatures were also maintained by the preservation of the high hole mobilities,^[^ ^31^ ^]^ leading to overall high *ZT*_avg, 300 – 473 K_ values, and better efficiency for generator or cooler. As shown in Figure 5b, this is higher as compared to the *ZT*_avg, 300 – 473 K_ values of many other reported Bi_y_Sb_2−y_Te_3_‐based composite materials. The measured *ZT* values at 373 K of the Bi_0.4_Sb_1.6_Te_3_‐Sb_2_Si_2_Te_6_ composites in this work were plotted against the reduced Fermi energy (*η*) together with the thermoelectric quality factor (*B*_TE_) analysis in Figure 5c, where the lines of various *B*_TE_ values were calculated based on the Single Parabolic Band (SPB) model (see Supporting Information for details). From the analysis, although the pristine *x* = 0% sample already seems to have an almost fully optimized *η* and reached its theoretical maximum *ZT*, the *x* = 1% sample was still able to achieve a much higher *ZT* due to its significantly larger *B*_TE_ of 0.64, as compared to that of the pristine sample (*B*_TE_ = 0.41). This implies that the small introduction of Sb_2_Si_2_Te_6_ nanoprecipitates is highly beneficial to the intrinsic transport properties of Bi_0.4_Sb_1.6_Te_3_ as the orderly atomic arrangement of the precipitate‐matrix interface region minimized the impact on the hole mobility, while the increased dislocation density in the interface region enabled a significant reduction of lattice thermal conductivity. Since *η* of the *x* = 1% sample was also very close to being fully optimized, not much improvements to the *ZT* can be expected from carrier concentration optimization alone. To explore the potential for maximizing *ZT*, by using the theoretical minimum lattice thermal conductivity (*κ*_L,min_), it is possible for *B*_TE_ to be improved to 1.02. Using the same *η* of the *x* = 1% sample, this would correspond to a high *ZT* value of 1.71. Such a high *ZT* may be achievable if this Sb_2_Si_2_Te_6_ compositing strategy is combined with other reported strategies, such as excess liquid Te sintering,^[^ ^12a^ ^]^ hot deformation,^[^ ^32^ ^]^ melt spinning,^[^ ^11^, ^12^, ^16^, ^33^ ^]^ aliovalent doping,^[^ ^3^, ^34^ ^]^ introducing porosity,^[^ ^8^, ^13^, ^15^, ^20^, ^34^ ^]^ entropy engineering and alloying with low thermal conductivity phases,^[^ ^34^, ^35^ ^]^ to further reduce *κ*_L_ to approach its theoretical minimum.

**Figure 6:**
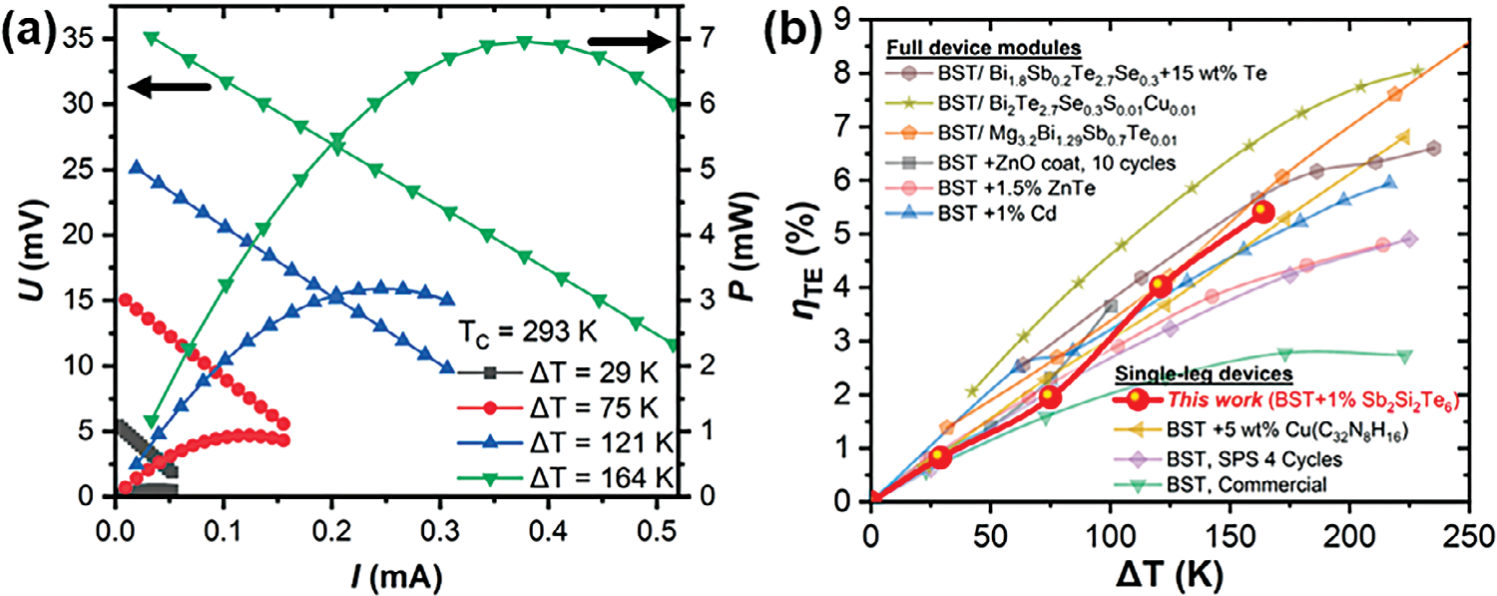
a) Current‐dependent output voltages and power of the *x* = 1% sample at various temperature gradients. b) Thermoelectric power conversion efficiencies (*η*_TE_) of the other reported Bi_y_Sb_2−y_Te_3_ (BST)‐based devices,^[^ ^12^, ^21^, ^36^, ^37^ ^]^ in comparison with the *x* = 1% (BST +1% Sb_2_Si_2_Te_6_) composite sample.

In order to assess the potential ability of the *x* = 1% sample to be used in thermoelectric device modules to harvest energy from low thermal gradient waste heat sources, a single‐leg device was fabricated from the same bar‐shaped sample after all the electrical resistivity and Seebeck coefficient measurements. From **Figure** **6**a, a maximum output power of 6.96 mW, which corresponds to a power density of 0.133 W cm^−2^, was measured from the single‐leg device at Δ*T* = 164 K. Thermoelectric power conversion efficiencies (*η*_TE_) of the *x* = 1% single‐leg device was plotted together with other reported BST‐based devices as a function of Δ*T* in Figure 6b. A peak *η*_TE_ of ≈5.4% was realized by the *x* = 1% single‐leg device at Δ*T* = 164 K, which is almost double the reported commercial BST single‐leg device efficiency (*η*_TE_ ≈ 2.8% at Δ*T* = 173 K),^[^ ^36^ ^]^ and is among the highest in that Δ*T* range as compared to the other reported BST‐based single‐leg devices.

## 3 Conclusion

In summary, we explored the effects of using an emerging thermoelectric material Sb_2_Si_2_Te_6_ as an additive in the Bi_0.4_Sb_1.6_Te_3_ matrix phase to produce Bi_0.4_Sb_1.6_Te_3_ + *x* mol% Sb_2_Si_2_Te_6_ composite materials. Through a gradual tuning of “*x*” from 0.5 to 4%, we found that our sample with an optimal composition of *x* = 1% has an improved electrical conductivity and reduced lattice thermal conductivity as compared to that of pristine Bi_0.4_Sb_1.6_Te_3_. Owing to the interface between the Bi_0.4_Sb_1.6_Te_3_ matrix and Sb_2_Si_2_Te_6_‐based precipitates having an orderly atomic arrangement and high dislocation density, phonons can be effectively scattered to a much greater degree than charge carriers, resulting in a successful enhancement of the peak *ZT* from 1.04 for the pristine sample to 1.35 for the *x* = 1% sample, at a temperature of 373 K. This demonstrates the importance of the interplay between the interface and the transport properties, as well as the synergistic nature of the strategy employed in this work, considering that pristine Sb_2_Si_2_Te_6_ has a reported *ZT* of 0.25 – 0.3 at the same temperature,^[^ ^24^ ^]^ which is significantly lower than that of pristine Bi_0.4_Sb_1.6_Te_3_. Moreover, the average *ZT* over a temperature range of 300 to 473 K was also enhanced from 0.88 for the pristine sample to a rather high value of 1.21 for the *x* = 1% sample. Finally, the high performance of the *x* = 1% sample was also evident with a high single‐leg device power conversion efficiency of ≈5.4% at Δ*T* = 164 K, which is among the highest in that Δ*T* range as compared to the other reported Bi_y_Sb_2−y_Te_3_‐based devices, making it a good candidate to be used in thermoelectric device modules to harvest energy from low thermal gradient waste heat sources.

## 4 Experimental Section

### Synthesis of Bi_0.4_Sb_1.6_Te_3_ and Sb_2_Si_2_Te_6_ Powders

Raw elements of Bi, Sb, Si, and Te (≥3N purity) were used in granular form. For the synthesis of pristine Bi_0.4_Sb_1.6_Te_3_, raw elements were weighed out according to the stoichiometric ratio and loaded into quartz tubes. The quartz tubes were flame‐sealed under high vacuum, transferred to a box furnace where the materials were heated to 1273 K in 8 h, held at that temperature for 12 h, and quenched in an ice bath. The resulting Bi_0.4_Sb_1.6_Te_3_ ingots were ground into powder by ball milling (SPEX 8000D) for 3 min, using a ball‐to‐material weight ratio of 2:1. For the synthesis of pristine Sb_2_Si_2_Te_6_, the raw elements were weighed out according to the stoichiometric ratio, loaded into ball milling jars, and milled (SPEX 8000D) for 1.5 h, using a ball‐to‐material weight ratio of 2:1. The powders were then loaded into quartz tubes, which were then flame‐sealed under high vacuum, transferred to a box furnace and heated to 823 K in 5 h, held at that temperature for 48 h, followed by natural cooling to room temperature. The resulting Sb_2_Si_2_Te_6_ ingots were ground into powder by ball milling (SPEX 8000D) for 1 h. For XRD analysis of the pristine Sb_2_Si_2_Te_6_ pellet, the ball‐milled Sb_2_Si_2_Te_6_ powder was sintered into a dense pellet according to the same conditions used by Luo et al.^[^ ^24^ ^]^ A discussion on the design rationale for ball milling durations was carried out and put in Supporting Information.

### Synthesis of Bi_0.4_Sb_1.6_Te_3_ + x mol% Sb_2_Si_2_Te_6_ Composite Pellets

The Sb_2_Si_2_Te_6_ powders were weighed out in various proportions according to *x* mol% of Bi_0.4_Sb_1.6_Te_3_ powders, for *x* = 1, 2, 3, 4. Each powder mixture was homogenized by ball milling (SPEX 8000D) for 1 h, using a ball‐to‐material weight ratio of 2:1, before loading the powders into graphite dies. The powders were then densified under vacuum at 753 K for 5 min by Plasma Activated Sintering (Ed‐PasIVJ, 6T‐3P‐30, Japan), under a uniaxial pressure of 60 MPa. The resulting composite pellets were all >97% of the theoretical density of Bi_0.4_Sb_1.6_Te_3_. It should be noted that the theoretical density of Sb_2_Si_2_Te_6_ is ≈82% of Bi_0.4_Sb_1.6_Te_3_’s theoretical density, causing the pellets to have decreasing densities with increasing *x*. The pellets were then cut into various shapes for subsequent measurements.

### Transport Property Measurements

All electrical and thermal transport measurements of the pellet samples were performed in the direction perpendicular to the pressing direction (in‐plane). A four‐point probe method (ULVAC‐RIKO ZEM‐3, Japan) was used to measure the electrical resistivities and Seebeck coefficients of bar‐shaped samples (11 × 2 × 2 mm^3^) from 300 to 473 K at every 25 K interval. Using the same temperature program, Laser Flash Apparatus (Netzsch LFA‐457, Germany) to obtain thermal diffusivity (*λ*) values from square‐shaped samples (10 × 10 × 1 mm^3^). Total thermal conductivity (*κ*) values were calculated by the equation *κ = λ·C_p_·D*, where the density (*D*) was measured by the Archimedes method. Since other reported works on pristine Bi_0.4_Sb_1.6_Te_3_ and pristine Sb_2_Si_2_Te_6_ show that neither material has any phase transition in the temperature range of 300 to 473 K, the specific heat capacity (*C_p_*) was calculated by the Dulong‐Petit limit, taking into account the precise composition and overall molecular weight of each different sample, as shown in Table S2 (Supporting Information). Calculated values of *κ*_e_ were derived from the Wiedemann–Franz relation *κ*_e_ = *L·σ·T*, where *L* is the Lorenz number calculated by the Single Parabolic Band (SPB) model. Commercial measurement instruments usually have percent errors of ±5% for electrical conductivity (σ), ±3% for Seebeck coefficient (*S*), and ±7% for thermal conductivity (*κ*).^[^ ^34a^ ^]^ The propagated percent error for power factor (PF) was determined to be ±7%, from the formula $\frac{d(PF)}{PF}=\sqrt{{\left(2\times \frac{dS}{S}\right)}^{2}+{\left(\frac{d\sigma }{\sigma }\right)}^{2}}\;$, while the propagated percent error for *ZT* was determined to be ±10% from the formula

$\;\frac{d(ZT)}{ZT}=\sqrt{{\left(2\times \frac{dS}{S}\right)}^{2}+{\left(\frac{d\sigma }{\sigma }\right)}^{2}+{\left(\frac{d\kappa }{\kappa }\right)}^{2}}\;$. Since the electronic contributions of thermal conductivities (*κ*_e_) were calculated from the Lorenz numbers (L), which were derived from the Seebeck coefficients, the propagated percent error for *κ*_e_ was determined to be ±6% from the formula $\frac{d(\kappa_{e})}{\kappa_{e}}=\sqrt{{\left(\frac{dS}{S}\right)}^{2}+{\left(\frac{d\sigma }{\sigma }\right)}^{2}}\;$. The propagated error for the lattice and bipolar thermal conductivities (*κ* − *κ*_e_) was determined from the formula $d\left(\kappa -\kappa_{e}\right)\;=\sqrt{{\left(d\kappa \right)}^{2}+{\left(d\kappa_{e}\right)}^{2}}\;$.

### Single‐leg Device Fabrication and Efficiency Measurement

After all electrical resistivity and Seebeck coefficient measurements, the same bar‐shaped sample was subsequently used as a single‐leg device, where a 100 nm layer of Ni was deposited by an electron‐beam evaporator (Denton Explorer, 5 × 10^−7^ Torr, evaporation rate of 1 Å s^−1^) on each end of the bar‐shaped sample. The sample was connected to the copper electrodes by applying InGa alloy on each end. The fabricated single‐leg device was then measured by the Mini‐PEM testing system (ULVAC‐RIKO, Japan). The current (*I*), voltage (*U*), and heat flow (*Q*) through the single‐leg device were measured directly, from which the output power (*P* = *U∙I*) and the power conversion efficiency (*η*_TE_ = *P*/*Q*) can be calculated.

### Structural Characterization

The overall phase purities of the Bi_0.4_Sb_1.6_Te_3_ + *x* mol% Sb_2_Si_2_Te_6_ samples were measured by Powder X‐ray Diffraction (XRD) analysis, using a Bruker D8‐Advance X‐ray powder diffractometer with Cu Kα radiation. A scan step of 0.02° from 2*θ* = 10° to 80° with a time per step of 0.17s was used for Figure 1a, while a scan step of 0.01° from 2*θ* = 43.3 – 44.2° with a time per step of 1.5s was used for Figure 1b. Microstructural and elemental analyses were performed by field‐emission scanning electron microscope (JEOL JSM 7600F) equipped with an Energy Dispersive X‐ray spectrometer (EDS) detector, as well as a transmission electron microscope (JEOL JSM‐2100). To ascertain the thermal stability of the composite samples, Differential Scanning Calorimetry (Mettler Toledo DSC 1) and Thermogravimetric Analysis (TA Instruments TGA Q500) measurements were performed.

## Conflict of Interest

The authors declare no conflict of interest.

## Author Contributions

X.Y.T., J.D., and J.L. contributed equally to this work. X.Y.T.: Conceived the ideas, prepared the samples, performed thermal measurements using LFA, data analysis, and wrote the manuscript. J.D.: Conceived ideas, electronic and thermal transport analysis, oversaw and supervised all structural characterization experiments and structural data analysis, and wrote the manuscript. J.L.: Performed HRTEM and STEM measurements, elemental analysis and other characterizations, and electrical property measurements, and edited the manuscript. D.Z. and S.F.D.S.: Assisted in XRD and SEM measurements and the required sample preparations. K.S., N.J., and I.J.W.J.Y.: Assisted in the material synthesis, electrical property measurements, and sample preparations. S.W.C. and X.W.: Assisted in the thermal property measurements and other sample preparations. L.H., Y.L., and Y.Z.: Advised on electronic and thermal transport analysis and sharing of first‐hand experience on handling Bi_0.4_Sb_1.6_Te_3_ and Sb_2_Si_2_Te_6_ materials. D.X.Y.S. and R.J.: Assisted in structural characterizations and sample preparations. K.C.H.G.: Assisted in preparation of a single‐leg device. Y.J. and J.‐F.L.: Performed single‐leg device efficiency measurements and analysis of results. A.S. and Z.Q.: Intellectual contribution to idea creation, experimental planning, thermal analysis, as well as both electronic and thermal transport analysis. J.X. and Q.Y.: Supervised and guided the entire work and provided timely advice on the experiments, edited the manuscript.

## Supporting information

Supporting Information

## Data Availability

The data that support the findings of this study are available from the corresponding author upon reasonable request.
