# Supplementary material for: Synergistic Combination of Sb2Si2Te6 Additives for Enhanced Average ZT and Single‐Leg Device Efficiency of Bi0.4Sb1.6Te3‐based Composites
Source: Adv Sci (Weinh). 2024 Mar 29;11(23):2400870. doi: 10.1002/advs.202400870 (PMC11187870; doi:10.1002/advs.202400870)
Supplement: Supplementary file 1 — Supporting Information [file ADVS-11-2400870-s001.pdf]

ADVANCED SCIENCE
Open Access

Supporting Information

for *Adv. Sci.*, DOI 10.1002/advs.202400870

Synergistic Combination of $Sb_2Si_2Te_6$ Additives for Enhanced Average ZT and Single-Leg Device Efficiency of $Bi_{0.4}Sb_{1.6}Te_3$-based Composites

Xian Yi Tan, Jinfeng Dong, Jiawei Liu, Danwei Zhang, Samantha Faye Duran Solco, Kıvanç Sağlık, Ning Jia, Ivan Joel Wen Jie You, Sheau Wei Chien, Xizu Wang, Lei Hu, Yubo Luo, Yun Zheng, Debbie Xiang Yun Soo, Rong Ji, Ken Choon Hwa Goh, Yilin Jiang, Jing-Feng Li, Ady Suwardi, Qiang Zhu, Jianwei Xu* and Qingyu Yan**

Supporting Information

Synergistic combination of $Sb_2Si_2Te_6$ additives for enhanced average ZT and single-leg device efficiency of $Bi_{0.4}Sb_{1.6}Te_3$-based composites

Xian Yi Tan[a,b#], Jinfeng Dong[b#*], Jiawei Liu[b,c#], Danwei Zhang[a], Samantha Faye Duran Solco[a], Kıvanç Sağlık[a,b], Ning Jia[b,d], Ivan Joel Wen Jie You[a,e], Sheau Wei Chien[a], Xizu Wang[a], Lei Hu[f], Yubo Luo[g], Yun Zheng[h], Debbie Xiang Yun Soo[a], Rong Ji[a], Ken Choon Hwa Goh[a], Yilin Jiang[i], Jing-Feng Li[i], Ady Suwardi[a,j], Qiang Zhu[a,c,k], Jianwei Xu[a,c,l*], Qingyu Yan[b*]

[#] These authors contributed equally

* Corresponding authors: jinfeng.dong@ntu.edu.sg; xu_jianwei@isce2.a-star.edu.sg; alexyan@ntu.edu.sg

Supporting Figures

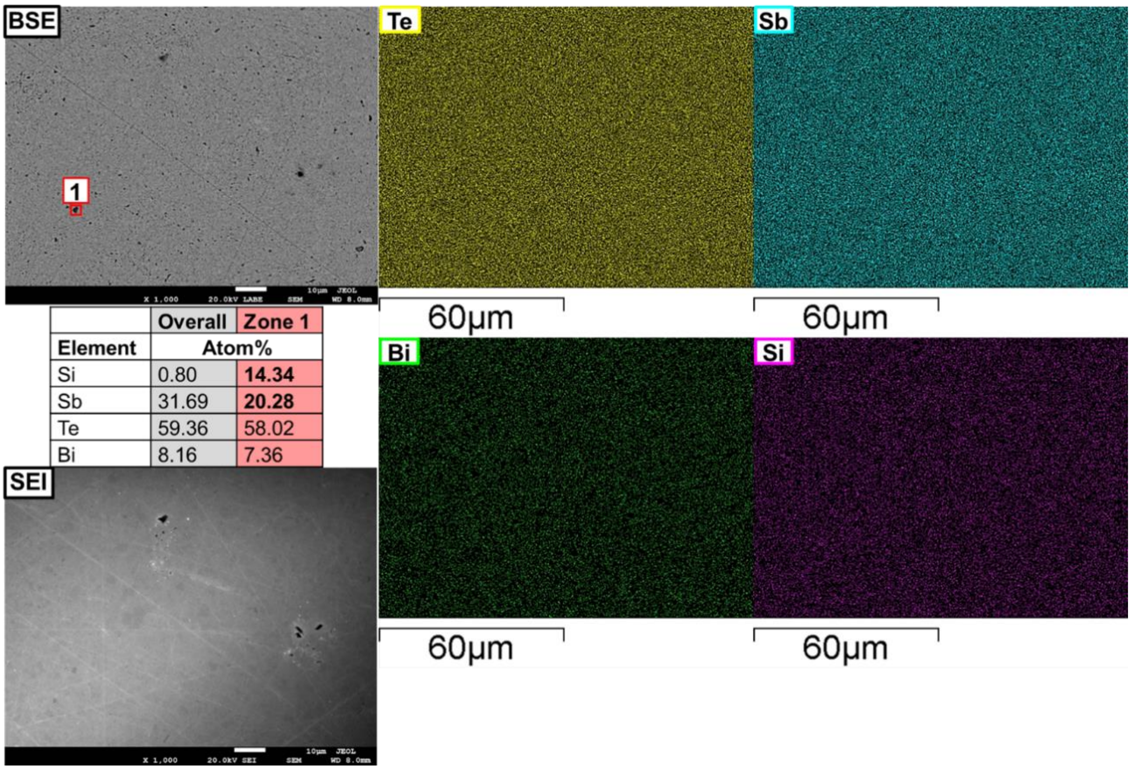

	Overall	Zone 1
Element	Atom%	
Si	0.80	14.34
Sb	31.69	20.28
Te	59.36	58.02
Bi	8.16	7.36

Figure S1. Elemental analysis of the x = 1% polished pellet sample by Scanning Electron Microscopy (SEM) elemental mapping and Energy Dispersive Spectroscopy (EDS). Backscattered Electron (BSE) image was taken to clearly identify regions of possible secondary phases, by comparisons with the Secondary Electron Image (SEI).

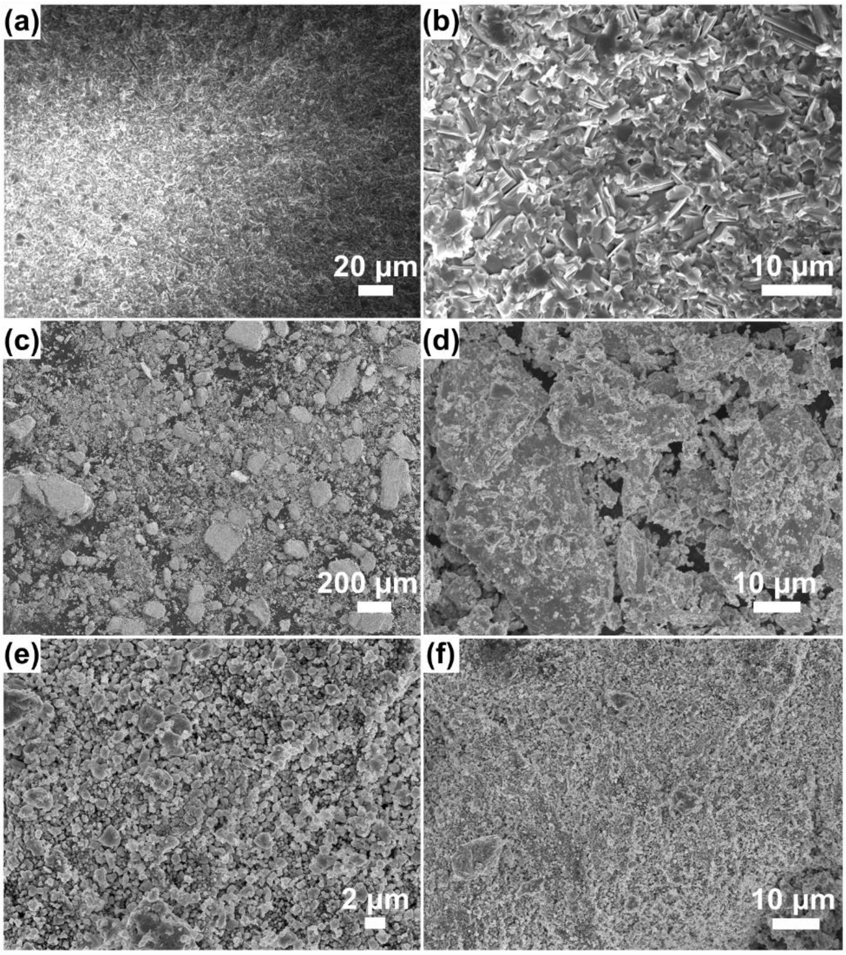

Figure S2. a) − b) SEM fractographic images of the x = 1% pellet sample. SEM images of c) − d) $Bi_{0.4}Sb_{1.6}Te_3$ powders after 3 min of ball milling and e) − f) $Sb_2Si_2Te_6$ powders after 1h of ball milling.

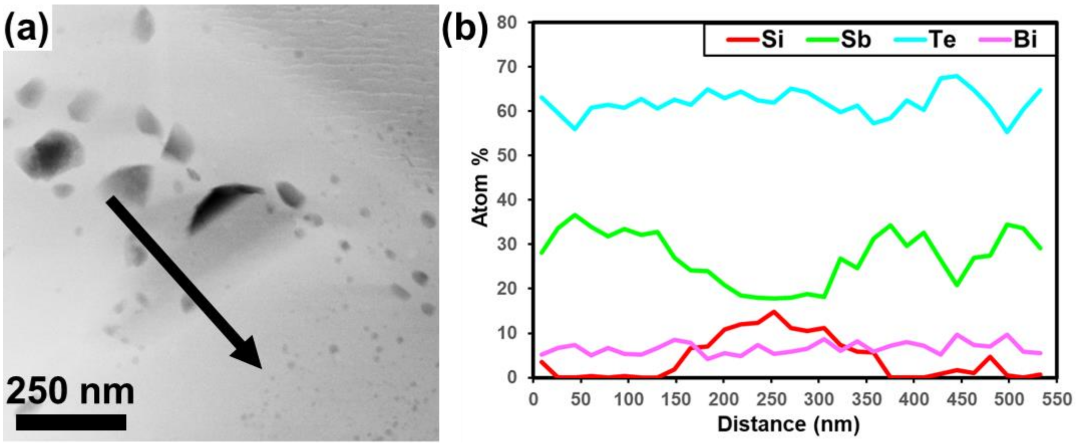

Figure S3. (a) Low magnification dark field STEM image of the x = 1% pellet sample. (b) EDS line scan profile of the elemental composition along the length and direction indicated by the black arrow in (a).

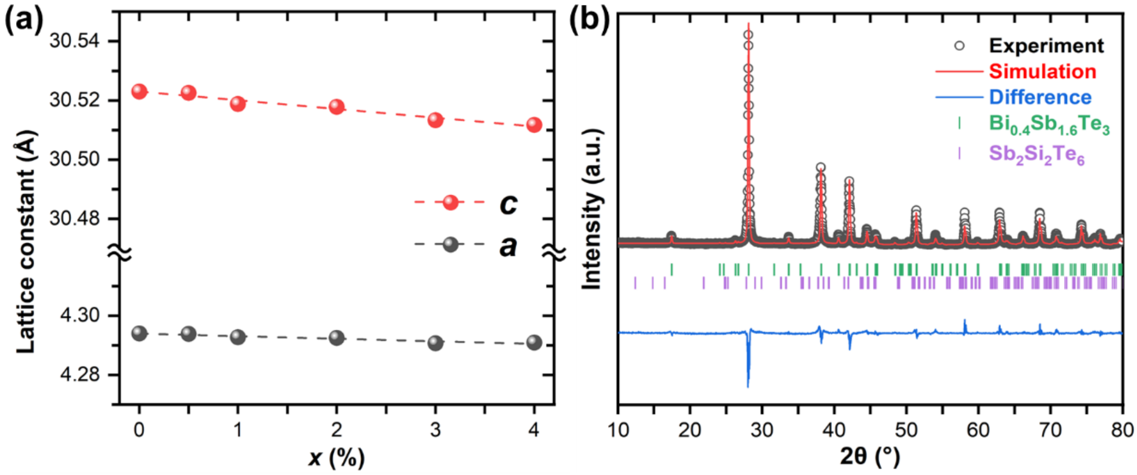

Figure S4. (a) Refined lattice constants of the $Bi_{0.4}Sb_{1.6}Te_3$ + *x* mol% $Sb_2Si_2Te_6$ samples. (b) Rietveld refinement pattern of the x = 1% sample.

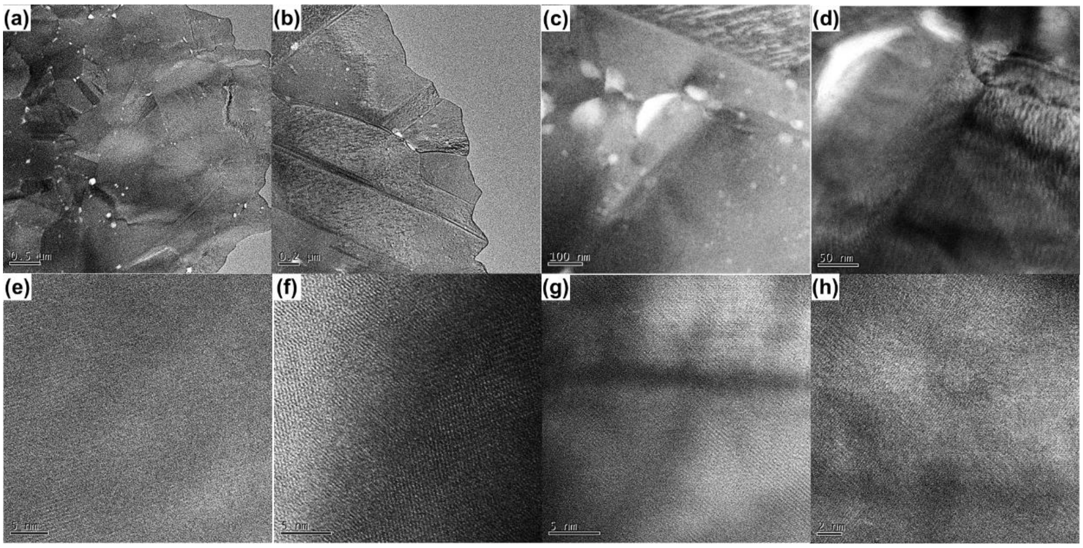

Figure S5. (a) – (d) Low magnification HRTEM images of the x = 1% polished pellet sample. High magnification HRTEM images of (e) $Sb_2Si_2Te_6$, (f) interface region 1, (g) interface region 2, and (h) $Bi_{0.4}Sb_{1.6}Te_3$.

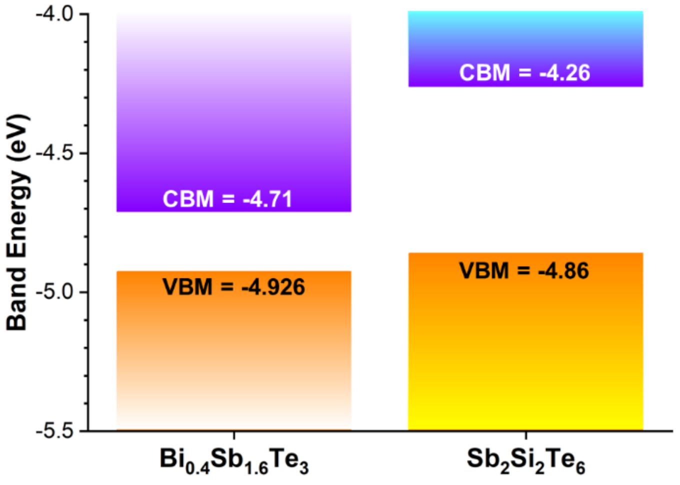

Figure S6. Valence band alignment between $Bi_{0.4}Sb_{1.6}Te_3$ and $Sb_2Si_2Te_6$, plotted based on valence band maxima (VBM) and conduction band minima (CBM), w.r.t. vacuum level.[1]

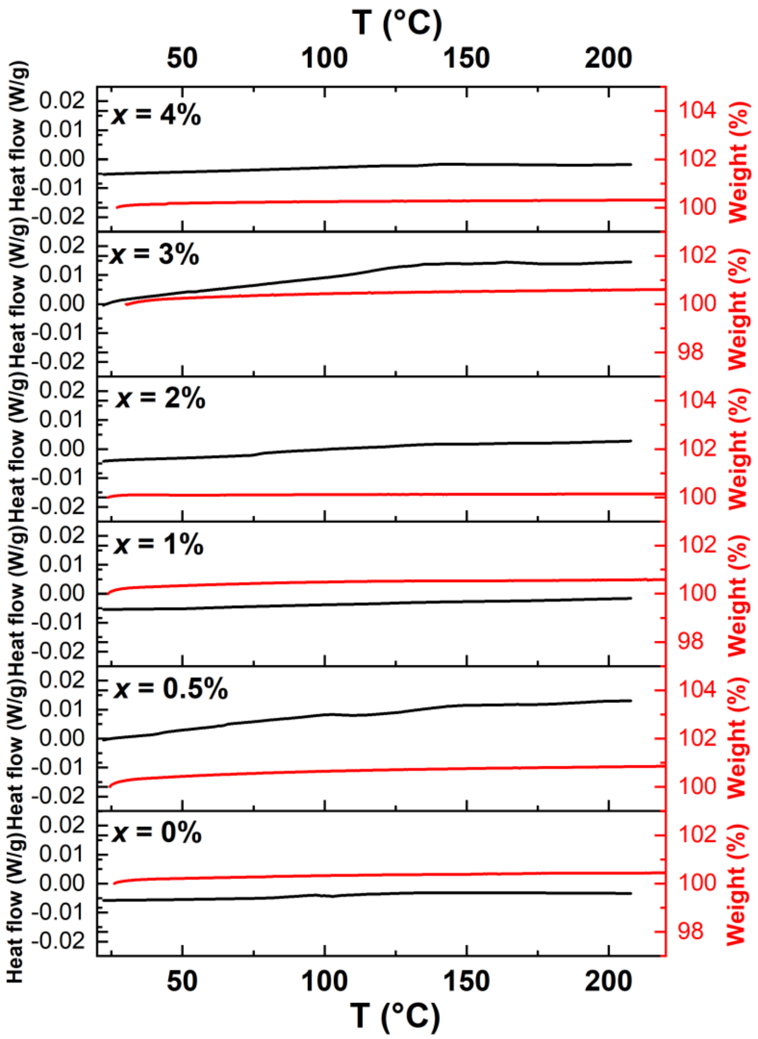

Figure S7. DSC (black) and TGA (red) measurements of the $Bi_{0.4}Sb_{1.6}Te_3$ + *x* mol% $Sb_2Si_2Te_6$ samples.

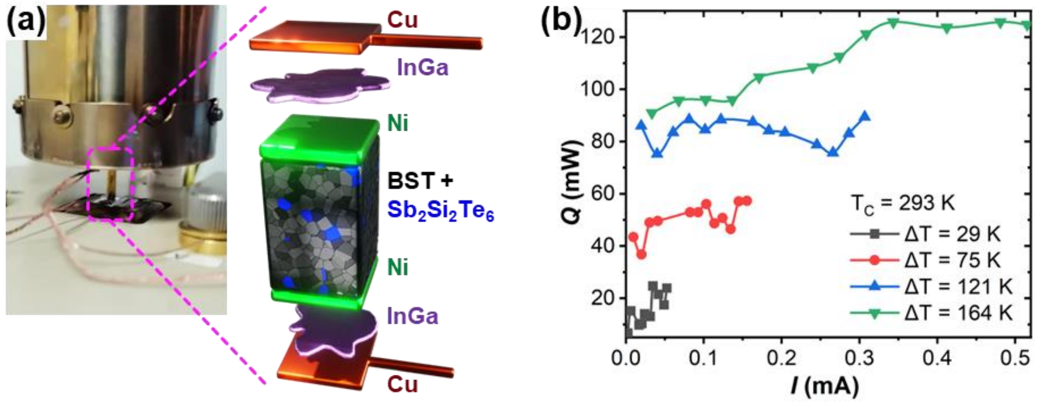

Figure S8. (a) Digital photograph of the loading of the single-leg device in the measurement equipment and a schematic for the electrode attachment on the bar-shaped x = 1% sample. (b) Measured heat flow (Q) of the x = 1% single-leg device as a function of current (I) at various temperature gradients.

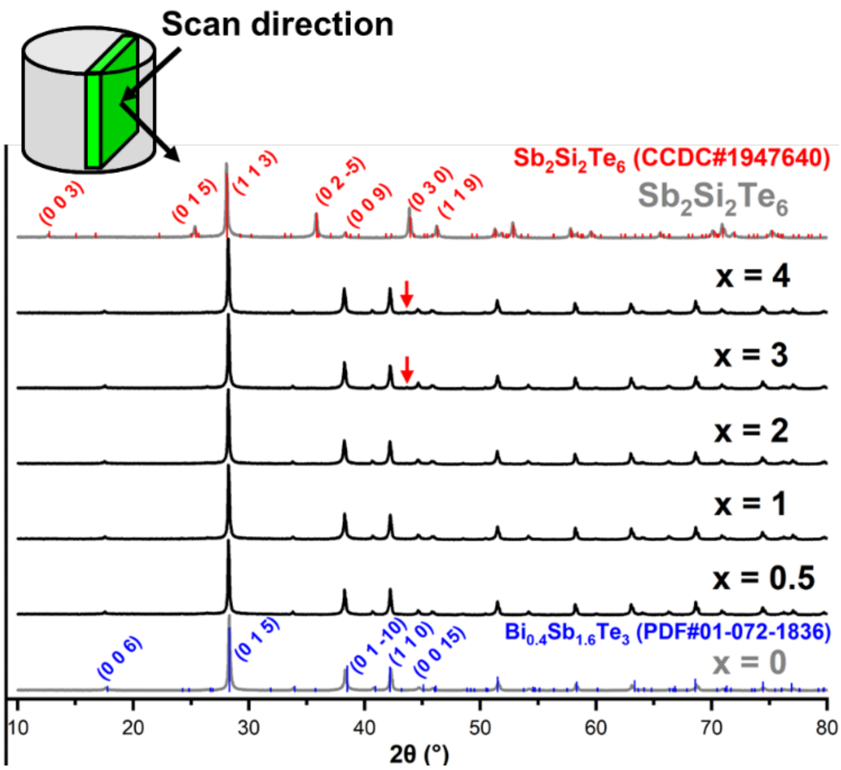

Figure S9. Powder X-ray diffractograms of $Bi_{0.4}Sb_{1.6}Te_3$ + x mol% $Sb_2Si_2Te_6$ pellet samples for varying amounts of x, scanned at 2θ = 10 – 80°, in the direction parallel to the pressing direction (cross-plane).

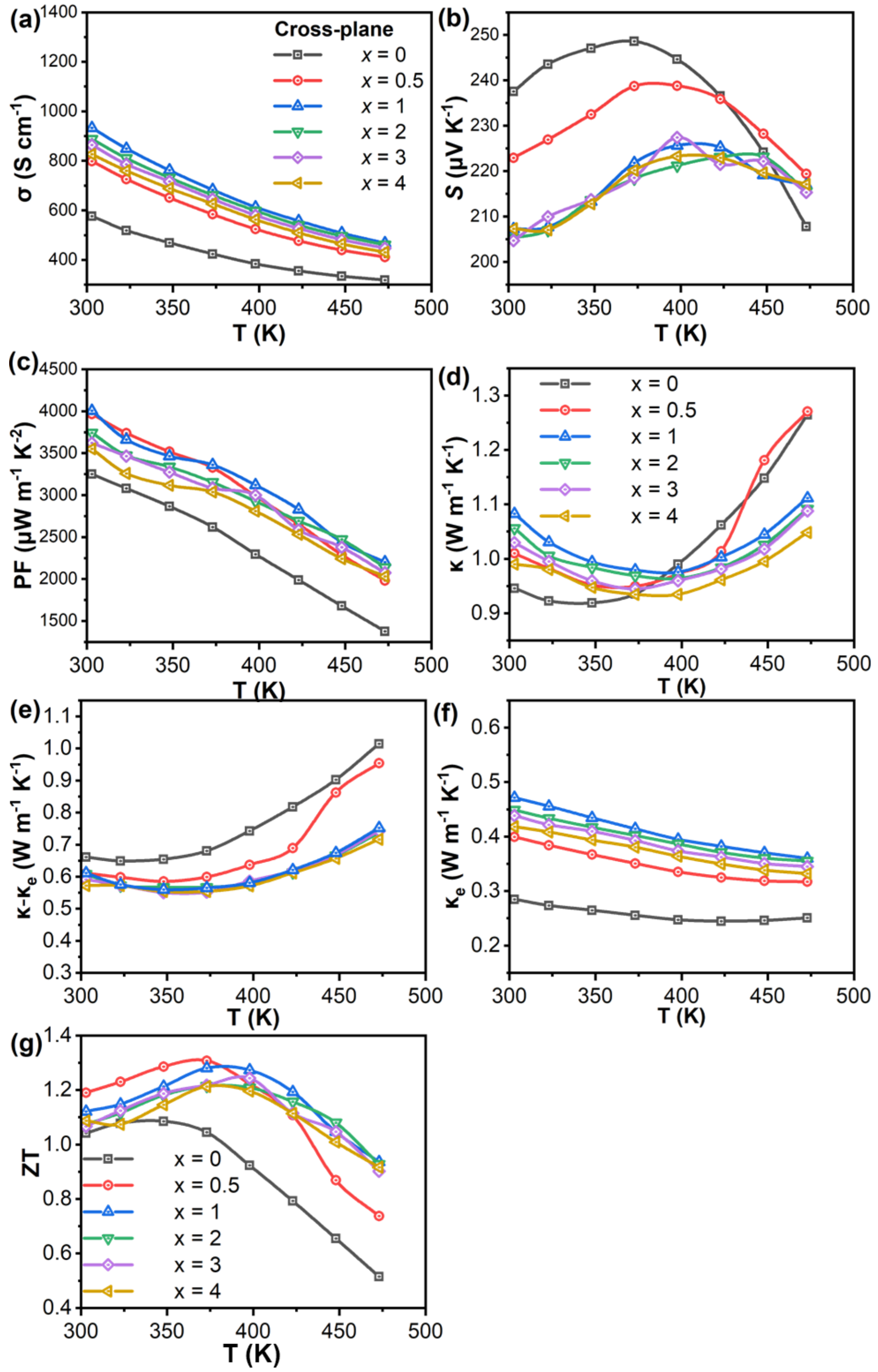

Figure S10. Temperature-dependent a) electrical conductivities (σ), b) Seebeck coefficients (*S*), c) thermoelectric Power Factors (PF), d) total thermal conductivities (κ), e) lattice and bipolar thermal conductivities ($\kappa - \kappa_e$), f) electronic contributions of thermal conductivities (κ_e), and g) thermoelectric Figures of Merit (*ZT*) of the composite samples, for various $Sb_2Si_2Te_6$ contents, measured in the direction parallel to the pressing direction (cross-plane).

Phase fraction analysis

To investigate the phase fraction of the composite samples, the XRD diffraction patterns of the composite samples were analysed by Rietveld refinement, where the experimentally measured patterns were fitted with $Bi_{0.4}Sb_{1.6}Te_3$ (PDF# 01-072-1836) and $Sb_2Si_2Te_6$ (CCDC# 1947640), using the FullProf Suite software, as shown in **Table S3**. In general, the phase fractions determined by Rietveld refinement were lower compared to the theoretical phase fractions determined based on the mol% of $Sb_2Si_2Te_6$ powders added to the $Bi_{0.4}Sb_{1.6}Te_3$ powders during the mixing step. In addition to the general errors associated with Rietveld refinement, such discrepancies may also be due to the $Sb_2Si_2Te_6$ peak intensity losses in the XRD patterns for the parts of the $Sb_2Si_2Te_6$ precipitates that were diffused with the matrix to from the interface region. However, such a diffusion is confined locally to the interface region between $Sb_2Si_2Te_6$ precipitates and the $Bi_{0.4}Sb_{1.6}Te_3$ matrix, therefore, it is not supposed to induce significant doping effects in $Bi_{0.4}Sb_{1.6}Te_3$ matrix as confirmed by the nearly unchanged lattice parameters shown in **Figure S4a**.

Design rationale for ball milling durations and particle sizes

For the rationale behind the milling durations, we aim for the $Sb_2Si_2Te_6$ powders to have a much smaller particle size than the $Bi_{0.4}Sb_{1.6}Te_3$ powders because we want a large proportion of the $Sb_2Si_2Te_6$ particles to be in the nano- or sub-micron size range for effective phonon scattering while having most of the $Bi_{0.4}Sb_{1.6}Te_3$ particles in the few-microns or tens-of-microns range in order to maintain the high hole mobility of the $Bi_{0.4}Sb_{1.6}Te_3$ matrix. Therefore, making a composite will involve 3 key milling steps:

Step 1: Pulverization of pre-synthesized BST ingots into powder

Step 2: Pulverization of pre-synthesized SST ingots into powder

Step 3: Mixing BST powders with SST powders

Before deciding on the milling durations for each material, we briefly reviewed the experimental conditions used in previous reports on BST composites. For the pulverization of pre-synthesized BST ingots into powder (**Step 1**), there is a very wide range of reported procedures, which can be as simple as hand grinding in a mortar and pestle for 5 min,[20] ball milling for 10 min,[21] or it can even be as thorough as ball milling for 24h.[22] Pulverization of pre-synthesized SST ingots into powder (**Step 2**) has always been performed by hand grinding in a mortar and pestle. In some of the few reports that were

found for mixing pre-synthesized BST ingots with a secondary phase (**Step 3**) using the SPEX ball milling machines, mixing can be performed for 0.5h,[18c] 1h,[23] or as long as 4h.[24]

Based on the reported information, the milling duration for the mixing (**Step 3**) was first decided to be performed for 1h, because we aim to mix the two different powders homogeneously but not longer than 1h so as to limit any mechanical alloying effects between the $Bi_{0.4}Sb_{1.6}Te_3$ and $Sb_2Si_2Te_6$ phases. In order to achieve a significantly smaller size of the $Sb_2Si_2Te_6$ particles as compared to the $Bi_{0.4}Sb_{1.6}Te_3$ particles, it was decided that the total milling time (throughout **Steps 1 – 3**) experienced by $Sb_2Si_2Te_6$ should be double that of $Bi_{0.4}Sb_{1.6}Te_3$.

Considering the additional 1h of ball milling at **Step 3**, we aim to limit the total milling duration of $Bi_{0.4}Sb_{1.6}Te_3$ to ~1h. Therefore, the initial pulverisation of the $Bi_{0.4}Sb_{1.6}Te_3$ ingots (**Step 1**) was only limited to 3 min, just for the purpose of barely breaking them down to an easily mixable form, to improve the homogeneity of the later mixing step (**Step 3**). A large amount of the $Bi_{0.4}Sb_{1.6}Te_3$ powders were observed to have particle sizes of >100 μm (**Figure S2c**) with some smaller particles of >10 μm (**Figure S2d**), after 3 min of milling.

In order to achieve a total milling duration of 2h for $Sb_2Si_2Te_6$, the initial pulverisation of the $Sb_2Si_2Te_6$ ingots (**Step 2**) was performed for 1h, which was enough to obtain a large amount of particles in the few-microns to sub-micron range as shown in **Figure S2e**. Therefore, it is expected that upon further milling of both powders for an additional 1h during the mixing process (**Step 3**), the few-micron sized particles of $Sb_2Si_2Te_6$ will be broken down into nano-sized powders, while the >100 μm sized particles of $Bi_{0.4}Sb_{1.6}Te_3$ will be broken down into particles in the few-microns or tens-of-microns range.

After undergoing the mixing process and sintering, the grain sizes of the resulting pellets were also analysed. From **Figures 1d, 2a** and **S1**, the average grain size of $Sb_2Si_2Te_6$ precipitates in the x = 1% composite sample is a few hundred nm. From **Figures S2a** and **S5a**, the average grain size of the $Bi_{0.4}Sb_{1.6}Te_3$ matrix range is a few microns. As for the relationship of the average grain size with the interface region, generally for the same total quantity of secondary phases, a smaller average grain size of secondary phases means more particles, which would lead to a higher total exposed surface area to form a higher total interface surface area with the matrix phase.

Single Parabolic Band (SPB) model

$$\mathrm{F_j}(\eta) = \int_0^\infty f\epsilon^{\mathrm{j}}\mathrm{d}\epsilon = \int_0^\infty \frac{\epsilon^{\mathrm{j}}\mathrm{d}\epsilon}{1+\exp(\epsilon-\eta)}$$

Firstly, the various steps in the SPB model employ the use of Fermi integrals (F_j), where the characteristic carrier scattering exponent (r) varies with the type of carrier scattering mechanism.[2] For all analyses in this work, the conduction of charge carriers is assumed to be predominantly limited by acoustic phonon scattering (r = -0.5). The reduced energy of the carrier (ϵ) is defined by $\epsilon = E \div k_BT$. The reduced Fermi energy (η) is defined by $\eta = E_F \div k_BT$ and can be treated as a form of charge carrier concentration, as it is also highly related to the Fermi energy. While the determination of charge carrier concentration requires Hall effect measurements, which may be affected by inaccuracies like non-linearity of the Hall voltage versus magnetic field, η can be directly calculated from the measured Seebeck coefficients (S) as shown below:

$$S(\eta) = \pm\frac{k_B}{e}\left[\eta - \frac{\left(r+\frac{5}{2}\right)F_{r+1.5}(\eta)}{\left(r+\frac{3}{2}\right)F_{r+0.5}(\eta)}\right]$$

As every material has a S value measured at various temperatures, the η value that corresponds to each S value can be used to find the Lorenz number (L) according to the equation below. This can be done for the S values at each temperature in order to obtain L at different temperatures to calculate κ_e across the entire temperature range.

$$L(\eta) = \left(\frac{k_B}{e}\right)^2\left[\frac{\left(r+\frac{7}{2}\right)F_{r+1.5}(\eta)}{\left(r+\frac{3}{2}\right)F_{r+0.5}(\eta)} - \left(\frac{\left(r+\frac{5}{2}\right)F_{r+1.5}(\eta)}{\left(r+\frac{3}{2}\right)F_{r+0.5}(\eta)}\right)^2\right]$$

For the thermoelectric quality factor (B_{TE}) analysis, various gradually increasing values of B_{TE} can be used to generate multiple curves of ZT against η, according to the equation below. The experimentally determined ZT values and their corresponding η can then be plotted as discrete points on the same graph in order to see which B_{TE} curve best matches the experimentally determined ZT value point, so as to estimate the B_{TE} value of a material at a particular temperature.

$$ZT(\eta) = \frac{S^2(\eta)}{\frac{(k_B/e)^2}{B_{TE}\cdot F_{r+0.5}(\eta)} + L(\eta)}$$

Simplified Debye-Callaway model for lattice thermal conductivity

Firstly, the average sound velocity (v_m) can be calculated from the longitudinal (v_L) and transverse (v_T) sound velocities by:

$$v_m = \left[\frac{1}{3}\left(\frac{1}{{v_L}^3} + \frac{2}{{v_T}^3}\right)\right]^{-\frac{1}{3}}$$

The acoustic branch maximum phonon frequency ($\omega_{max,a}$) can then be determined from the number of atoms in primitive cell (N_{cell}) and the average atomic volume (Ω) of the matrix by:

$$\omega_{max,a} = \frac{\omega_D}{{N_{cell}}^{1/3}} = \left(\frac{6\pi^2}{\Omega \cdot N_{cell}}\right)^{1/3} \cdot v_m$$

The acoustic branch Debye temperature ($\theta_{D,a}$) can then be expressed as:

$$\theta_{D,a} = \frac{\hbar \cdot \omega_{max,a}}{k_B}$$

Subsequently, the total phonon scattering rate (τ^{-1}) can be calculated by summing up the contributions from various phonon scattering processes such as Umklapp (${\tau_U}^{-1}$), grain boundaries (${\tau_{GB}}^{-1}$), point defects (${\tau_{PD}}^{-1}$), nanoprecipitates (${\tau_{NP}}^{-1}$), dislocation core (${\tau_{DC}}^{-1}$) and strain (${\tau_{DS}}^{-1}$), through the Matthiessen's Rule:

$$\tau^{-1} = {\tau_U}^{-1} + {\tau_{GB}}^{-1} + {\tau_{PD}}^{-1} + {\tau_{NP}}^{-1} + {\tau_{DC}}^{-1} + {\tau_{DS}}^{-1}$$

The Umklapp phonon-phonon scattering rate (${\tau_U}^{-1}$) can be calculated from the average atomic mass of the matrix ($\bar{M}$), Grüneisen parameter (γ_G), and the comprehensive coefficient between Umklapp and Normal phonon-phonon scattering processes (A_N), as shown below:

$$\tau_U^{-1} = A_N \cdot \frac{2}{(6\pi^2)^{1/3}} \cdot \frac{k_B \Omega^{1/3} {\gamma_G}^2 \omega^2 T}{\bar{M} {v_m}^3}$$

The grain boundary contribution to the phonon scattering rate (${\tau_{GB}}^{-1}$) can be calculated from the average grain size (d) by:

$$\tau_{GB}^{-1} = \frac{v_m}{d}$$

The point defect contribution to the phonon scattering rate (τ_{PD}^{-1}) can be determined as shown below:

$$\tau_{PD}^{-1} = \frac{\Omega\omega^4}{4\pi v_m{}^3} \cdot \Gamma = \frac{\Omega\omega^4}{4\pi v_m{}^3} \cdot \left[\sum_i f_i \left(1 - \frac{m_i}{\bar{m}}\right)^2 + \sum_i f_i \left(1 - \frac{r_i}{\bar{r}}\right)^2\right]$$

The point defect scattering parameter (Γ), which represents the mass and strain contrast induced in the lattice from the point defects, consists of other variables. On an atomic site with average mass $\bar{m}$ and radius $\bar{r}$, f_i represents the fraction of dopant atoms with mass m_i and radius r_i. Therefore, the larger the differences of m_i from $\bar{m}$ and r_i from $\bar{r}$ respectively, the greater the mass and strain contrast.

The nanoprecipitate contributions to the phonon scattering rate (τ_{NP}^{-1}) can be calculated from the average radius of the precipitates (R), number density of the precipitates (N_{NP}), mass density of the matrix (D) and the density difference between the matrix and the precipitate (ΔD), as shown below:

$$\tau_{NP}^{-1} = v_m \cdot N_{NP} \cdot \left[(2\pi R^2)^{-1} + \left(\pi R^2 \frac{4}{9}\left(\frac{\Delta D}{D}\right)^2 \left(\frac{\omega R}{v_m}\right)^4\right)^{-1}\right]^{-1}$$

The dislocation contributions to the phonon scattering rate can be separated into dislocation core (τ_{DC}^{-1}) and dislocation strain (τ_{DS}^{-1}) scattering, which can be determined from the number density of dislocations (N_D), effective Burger's vector (B_D), Poisson's ratio (r_P), Grüneisen parameter (γ) and its change due to the dislocation strain (Δγ), as shown below:

$$\tau_{DC}^{-1} = N_D \cdot \frac{\Omega^{4/3}}{v_m{}^2} \cdot \omega^3$$

$$\tau_{DS}^{-1} = 0.6 \times B_D^2 N_D (\gamma_G + \Delta\gamma)^2 \omega \left[\frac{1}{2} + \frac{1}{24}\left(\frac{1 - 2r_P}{1 - r_P}\right)^2 \left\{1 + \sqrt{2}\left(\frac{v_L}{v_T}\right)^2\right\}^2\right]$$

The spectral heat capacity $C_s(\omega)$ can be expressed as:

$$C_s(\omega) = \frac{3k_B\omega^2}{2\pi^2 v_m{}^3}$$

By assuming the phonon group velocity remains constant and approximately equal to the average sound velocity (v_m), we can express the spectral lattice thermal conductivity $\kappa_s(\omega)$ as:

$$\kappa_s(\omega) = C_s(\omega){v_m}^2\tau(\omega)$$

Finally, the lattice thermal conductivity can be obtained by integrating the spectral thermal conductivity over the entire frequency range up to $\omega_{max,a}$, as shown below:

$$\kappa_L = \frac{1}{3}\int_0^{\omega_{max,a}} C_s(\omega)v^2\tau(\omega)\,d\omega$$

Debye-Cahill model for minimum lattice thermal conductivity ($\kappa_{L,min}$)

Assuming the material has a completely disordered or amorphous structure, the Debye-Cahill model can be used to predict the theoretical minimum lattice thermal conductivity ($\kappa_{L,min}$) or the amorphous limit,[3] as shown in the formulas below:

$$\kappa_{L,min} = \left(\frac{\pi}{6}\right)^{1/3} k_B {n_a}^{2/3} \sum_i v_i \left(\frac{T}{\theta_i}\right)^2 \int_0^{\theta_i/T} \frac{x^3 e^x}{(e^x - 1)^2}\,dx$$

Where:

$$\theta_i = v_i\left(\frac{\hbar}{k_B}\right)6\pi^2 n_a$$

$$x = \frac{\hbar\omega}{k_B T}$$

Supporting Tables

Table S1. Input parameters used for the Debye-Callaway and Debye-Cahill models.

Parameters	Symbols	Values	Source
Average atomic volume of $Bi_{0.4}Sb_{1.6}Te_3$	Ω	3.23×10^{-29} m^3	Ref. [4]
Average atomic volume of Sb_2Te_3	Ω_S	3.13×10^{-29} m^3	Ref. [5]
Average atomic volume of Bi_2Te_3	Ω_B	3.40×10^{-29} m^3	Ref. [5]
Number density of atoms in $Bi_{0.4}Sb_{1.6}Te_3$	n_a	3.09×10^{28} m^{-3}	Calculated
Longitudinal sound velocity	v_L	3030.62 m/s	Ref. [6]
Transverse sound velocity	v_T	1687.55 m/s	Ref. [6]
Average sound velocity	v_m	1879.17 m/s	Calculated
Number of atoms in primitive cell of $Bi_{0.4}Sb_{1.6}Te_3$	N_{cell}	5	Ref. [7]
Acoustic branch maximum frequency	$\omega_{max,a}$	13.44 THz	Calculated
Acoustic branch Debye temperature	$\theta_{D,a}$	102.7 K	Calculated
Average atomic mass of $Bi_{0.4}Sb_{1.6}Te_3$	$\bar{M}$	2.20×10^{-25} kg	Ref. [4]
Average atomic mass of Sb_2Te_3	$\bar{M}_S$	2.08×10^{-25} kg	Ref. [4]
Average atomic mass of Bi_2Te_3	$\bar{M}_B$	2.66×10^{-25} kg	Ref. [4]
Poisson's ratio	r_P	0.275	Calculated
Grüneisen parameter	γ_G	1.629	Calculated
Comprehensive coefficient between Umklapp and Normal processes	A_N	2.6	Ref. [4]
Average grain size	d	2 μm	Experimental
Point defect scattering parameter	Γ	0.21	Fitted
Mass density of $Bi_{0.4}Sb_{1.6}Te_3$ (host)	D	6.7932 g/cm^3	Ref. [4]
Mass density difference between $Bi_{0.4}Sb_{1.6}Te_3$ and $Sb_2Si_2Te_6$	ΔD	1.1622 g/cm^3	Calculated
Average effective mean radius of the nanoprecipitates	R	612.5 nm	Experimental
Number density of the nanoprecipitates	N_{NP}	1.61×10^{17} m^{-3}	Experimental
Magnitude of Burger's vector	B_D	12.7 Å	Ref. [4]
Bulk modulus of Sb_2Te_3	K	44.8 GPa	Ref. [5]
Sintering temperature	T_a	753 K	Experimental
Number density of the dislocations	N_D	1.3×10^{15} m^{-2}	Fitted

Table S2. Precise nominal compositions, molecular weights (M.W., based on 100 atoms), calculated specific heat capacity values (C_p), calculated theoretical densities, actual measured densities and their percentages relative to the calculated theoretical densities, for all $Bi_{0.4}Sb_{1.6}Te_3$ + *x* mol% $Sb_2Si_2Te_6$ samples. The *x* = ∞% sample represents the pristine $Sb_2Si_2Te_6$.

x (%)	Nominal composition (%)				M.W. (g/mol)	C_p ($J g^{-1} K^{-1}$)	Density ($g cm^{-3}$)		Relative Density (%)
	Bi	Sb	Te	Si			Theoretical	Actual	
**0**	8.00	32.00	60.00		13224.16	0.1886	6.790	6.712	98.85
**0.5**	7.92	31.88	60.00	0.20	13198.71	0.1890	6.784	6.689	98.60
**1**	7.84	31.76	60.00	0.39	13173.75	0.1893	6.779	6.664	98.31
**2**	7.69	31.54	60.00	0.77	13125.27	0.1900	6.767	6.655	98.34
**3**	7.55	31.32	60.00	1.13	13078.62	0.1907	6.756	6.639	98.27
**4**	7.41	31.11	60.00	1.48	13033.70	0.1914	6.745	6.608	97.96
∞		20.00	60.00	20.00	10652.91	0.2341	5.631		

Table S3. Phase fraction of $Sb_2Si_2Te_6$ in the $Bi_{0.4}Sb_{1.6}Te_3$ + *x* mol% $Sb_2Si_2Te_6$ composite samples.

x (%)	Nominal composition (mol% of $Sb_2Si_2Te_6$)	Weight % of $Sb_2Si_2Te_6$	
		Theoretical	Refinement
**0.5**	0.498	0.80	0.06
**1**	0.990	1.58	0.30
**2**	1.961	3.12	0.94
**3**	2.913	4.60	2.03
**4**	3.846	6.04	3.21

References

[1] a)Y. Luo, S. Cai, S. Hao, F. Pielnhofer, I. Hadar, Z.-Z. Luo, J. Xu, C. Wolverton, V. P. Dravid, A. Pfitzner, Q. Yan, M. G. Kanatzidis, *Joule* **2020**, 4, 159; b)W. H. Shin, J. W. Roh, B. Ryu, H. J. Chang, H. S. Kim, S. Lee, W. S. Seo, K. Ahn, *ACS Applied Materials & Interfaces* **2018**, 10, 3689.

[2] A. F. May, G. J. Snyder, in *Materials, preparation, and characterization in thermoelectrics*, CRC press, 2017.

[3] D. G. Cahill, S. K. Watson, R. O. Pohl, *Physical Review B* **1992**, 46, 6131.

[4] H.-L. Zhuang, H. Hu, J. Pei, B. Su, J.-W. Li, Y. Jiang, Z. Han, J.-F. Li, *Energy & Environmental Science* **2022**, 15, 2039.

[5] G. Yang, R. Niu, L. Sang, X. Liao, D. R. G. Mitchell, N. Ye, J. Pei, J.-F. Li, X. Wang, *Advanced Energy Materials* **2020**, 10, 2000757.

[6] J. Pei, H. Li, H.-L. Zhuang, J. Dong, B. Cai, H. Hu, J.-W. Li, Y. Jiang, B. Su, L.-D. Zhao, J.-F. Li, *InfoMat* **2022**, n/a, e12372.

[7] D. Bessas, I. Sergueev, H. C. Wille, J. Perßon, D. Ebling, R. P. Hermann, *Physical Review B* **2012**, 86, 224301.
